# The status quo and evolution of glycoconjugates for cancer immunotherapy

**DOI:** 10.1007/s10719-026-10227-z

**Published:** 2026-10-28

**Authors:** Yashapal Singh, Mohini Kamra

**Affiliations:** 1 Department of Chemical Engineering, University of Texas at Austin, Austin, TX 78712 USA; 2 Department of Biomedical Sciences, University of South Alabama, Mobile, AL 36688 USA

**Keywords:** Cancer immunotherapy, Cancer vaccine, Anti-tumor immune response, Glycan, Polysaccharide, Glycoconjugate, Lectin receptors, Carbohydrate antigen, Aberrant glycosylation, TACAs

## Abstract

Glycans and glycoconjugates play a significant role in nearly all aspects of living organisms from birth through development to demise. For more than half a century, the aberrant glycosylation profiles on tumor cells have been discussed. However, in the past few decades scientists have validated how these modified glycans influence cancer development, progression and metastasis through bypassing immune surveillance and creating an immunosuppressive milieu in the tumor microenvironment. Although advancements in cancer immunotherapy have shown promise, it has only been able to enhance the overall survival of a small group of patients. Certain types of cancer remain undiagnosed and untreated until very late stages, due to the limited understanding of complex tumor immune resistance. The understanding and use of glycans and glycoconjugates help researchers to work towards broadening the benefits of cancer immunotherapy to a larger population. Many glycans and glycoconjugates have made their way to clinical trials but still the full potential of glycan-based materials has not been entirely harnessed. This comprehensive review outlines major developments in the field of glycoconjugate mediated cancer immunotherapy, with special emphasis on the most recent developments. While covering a wide range of glycan categories, attempts have been made to draw correlations between glycoconjugate structure and the primary immune response elicited by each of those glycan categories.

## Introduction

### Introduction to glycans and glycoconjugates implicated in cancer

Carbohydrates are crucial to all living organisms due to their fundamental use as energy sources and as components of medicinal products. Glycans, the sugar part of glycoproteins and glycolipids, encompass a vast structural diversity and play distinctive roles in a series of biological events such as metabolism, development, immune response, pathogenesis, and disease abrogation [1]. Through the glycan component, glycoproteins and glycolipids participate in interactions at both cellular and non-cellular levels. The identification of tumor-associated glycoproteins such as α-fetoprotein (AFP), carcinoembryonic antigen (CEA), and human epidermal growth factor receptor 2 (HER2) steered the early development of glycan-based biomarkers in the 1960’s [2, 3].

The role of glycans in tumor biology was first discovered in the early 20th century, wherein truncated glycans were detected at cell surface proteins [4, 5]. One of the concurrent seminal discoveries established that dysregulation of glycosyltransferases and glycosidases in cancer drives glycan truncation on cancer cell surfaces [6]. Since then, glycans and glycoconjugates have received researchers’ attention with the aim of understanding their role in cancer development and for their theranostic potential. The identification of altered glycans on tumor cells, compared to healthy cells was a pivotal discovery in the 1950’s [4] and these altered glycan structures were categorized as “tumor-associated carbohydrate antigens (TACAs)”. Since the genesis of research on cancer glycome, TACAs have been proven to contribute to tumorigenesis and cancer progression by facilitating immune escape, proliferation, invasion and metastasis [7–13]. There is a list of well-identified and characterized TACAs in various cancer types, including truncated *O*-glycans such as Thomsen-Nouveau (Tn), Thomsen-Friedreich (TF), sialyl-Tn (sTn), gangliosides (GD2, GD3, GM2, GM3 and fucosyl-GM1), Sialyl Lewis antigens (sLe^a^, sLe^x^ and sLe^y^), Globo series (Globo-H, SSEA-3 and SSEA-4) and polysialic acid [14–17]. Because these are “self” antigens, they are often more difficult for the immune system to detect and target. A pioneering approach targeting these cancer-associated molecular signatures was the development of TACA-targeted monoclonal antibodies (mAbs) [18, 19]. While GD2-targeting antibodies (e.g., dinutuximab) were developed for neuroblastoma [20, 21] and anti-MUC1 antibodies were explored for breast and ovarian cancers [22], a major turning point came with the development of synthetic carbohydrate-based vaccines, using multivalent glycoconjugate approaches.

Growing evidence over decades has established that TACAs actively foster a hostile immunosuppressive tumor microenvironment by interacting with lectins present on the surface of immune cells [23]. Lectins are a class of carbohydrate-binding proteins that bind specific sugar moieties and have proven to be indispensable tools in deciphering the cancer glycome [24]. Due to their specific binding abilities, lectins are tailored as diagnostic markers, biosensors and even therapeutic agents [25]. The ability of lectins to differentiate tumors from normal tissues relies on their glycosylation patterns and has established them as an invaluable cancer diagnostic tool [26]. In recent years, lectins have emerged as targeted drug delivery agents, aiding the design and development of glycan-targeted immunotherapies with enhanced treatment efficacy [23, 26]. The lectins which have commonly been targeted for therapeutic purposes include Galectins (β-galactoside binding lectins), Siglecs (Sialic acid-binding immunoglobulin-like lectins), and C-type lectin receptors (CLRs) [27–29]. While galectins are overexpressed on cancer cells, Siglecs and CLR’s are overexpressed on the surface of the tumor-associated immune cells. The knowledge of these three classes of glycan-binding proteins lays the foundation for designing immunotherapeutic interventions using glycans and glycoconjugates [30, 31].

The overexpression of carbohydrate-binding receptors on the surface of cancer cells and tumor-associated immune cells has informed research on the use of different sugar-guided conjugates for delivery of cytotoxic payloads. Specifically, glucose transporter 1 (GLUT1), Cluster of differentiation 44 (CD44) as well as certain lectins like Siglecs and CLRs have been predominant targets of monosaccharide/polysaccharide-based therapeutic agents [32, 33]. Although the development of the field of glycoconjugates has seen some consolidations in recent years [34], glycoconjugate-based cancer immunotherapies have been rapidly evolving. Hence, the convergence of advancements in this specific area needs a dedicated synopsis. Presented below is a comprehensive overview of the many glycans and glycoconjugates developed for cancer immunotherapy. In this review, only those sugars and sugar conjugates are exclusively highlighted which trigger an anti-tumor immune response.

### Modes of action of cancer immunotherapy mediated by glycoconjugates

**Fig. 1:**
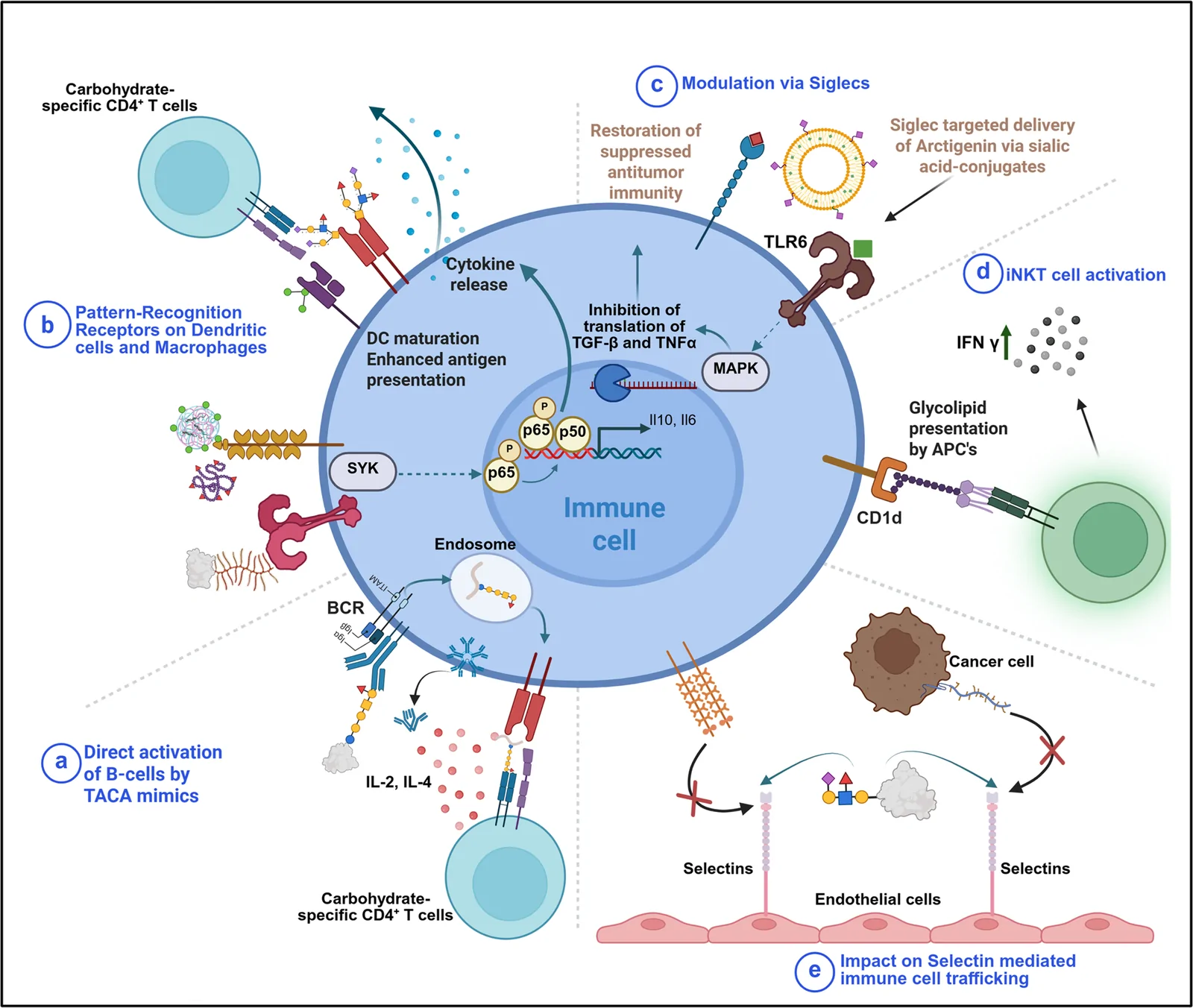
Mechanistic representation of anticancer immune responses elicited by glycans and glycoconjugates. The generic immune cell at the center represents DC’s, macrophages or B-cells in the respective sections, indicating specific immune cell receptors participating in these interactions

Cancer immunotherapy revolves around harnessing the potential of the body’s own immune system to fight cancer. It involves strategies to enhance the immune system’s ability to recognize and destroy cancer cells, thereby conferring greater specificity and higher durability of treatment [35]. At present, there are five main strategies that comprise cancer immunotherapy: (i) monoclonal antibodies and Bispecific T-cell engagers (BiTEs), (ii) immune checkpoint inhibitors, (iii) adoptive cell transfer, (iv) cancer vaccines and (v) oncolytic viruses [36–38]. Owing to their preferential expression on cancer cells and instrumental role in oncogenesis [23], TACAs have laid a solid foundation for the development of prophylactic and therapeutic vaccines for combating cancer [39]. The development of glycan/glycoconjugate-based vaccines and other immunotherapeutic approaches requires a thorough understanding of how TACAs contribute to immune evasion. The interactions of sugar-based systems with immune cells occur through diverse mechanisms that have been schematically depicted in Fig. 1. The physical presentation of a glycan is the primary driver of its immunological fate and the diverse mechanistic rationales, as supported by extensive experimental data, are detailed below:

- **Direct Activation of B-cells by TACA mimics**: Solitary or simple TACAs, such as isolated Tn or TF antigens, are functionally invisible to the immune system. Their low valence prevents receptor cross-linking and fails to recruit the cellular help required for activation. Some specific TACAs such as the highly repetitive or clustered sequences for example, mucins like MUC1, can directly cross-link B-cell receptors (BCRs), and induce a rapid B-cell activation identified as a “primitive” reaction [40]. This typically results in transient IgM responses and these TACAs are classified as T-cell-independent (TI) type 2 antigens [41]. This TI-2 pathway is dominated by IgM antibodies and lacks the somatic hypermutation required for high-affinity binding or the generation of long-term immunological memory. When TACA mimics are conjugated to carrier proteins these responses are enhanced by isotype switching to IgG and the formation of immunological memory [42, 43]. The underlying chemical structure of the carrier to which a glycan is tethered determines the “flavor” of the resulting immune response, i.e. whether the environment becomes pro-inflammatory or suppressive. One of the most notable carrier proteins is Keyhole Limpet Hemocyanin (KLH) whose conjugation to TACA units facilitates the recruitment of T-cell help to the initial B-cell response, providing the necessary co-stimulation to break tolerance (Fig. 1a). When the immune system processes this TACA-carrier complex, it facilitates the induction of IgG1 and IgG3, the isotypes which are the most effective at mediating tumor clearance.
- **Engagement of Pattern Recognition Receptors (PRRs)**: PRRs are specialized receptors on many innate immune cells that recognize Pathogen-Associated Molecular Patterns (PAMPs) as motifs of pathogen/non-self-recognition [44]. In line with many other sugar-based PAMPs, tumor glycans and their mimics are capable of activating these PRRs on macrophages, dendritic cells, and antigen presenting cells [45]. PRRs that have been the primary focus in the context of cancer immunotherapy are C-type lectin receptors (CLRs) and Toll-like receptors (TLRs), both of which recognize specific carbohydrate structures [46].

CLRs recognize fucose- and mannose-containing glycans using their C-type lectin-like domain (CTLD) that facilitates calcium-dependent glycan binding [47]. Post activation, CLRs (e.g., DC-SIGN, Langerin, Dectin-1) modulate immune responses in different ways via specific motifs in their cytoplasmic domains [48]. Based on their signaling properties, CLRs are classified into activating CLRs (containing immunoreceptor tyrosine-based activation motifs or ITAMs) and inhibiting CLRs (containing immunoreceptor tyrosine-based inhibitory motifs or ITIMs) [49–51]. Broadly, CLRs act as gatekeepers of immune surveillance by recognizing specific sugar structures [52, 53]. Sugar recognition triggers the modulation of innate and adaptive immunity [54]. Taking advantage of specific glycan recognition, researchers have targeted CLRs by designing glycomimetics [55]. Interestingly, the aberrant expression of some CLRs (like CD205) on specific cancer cells has also been reported [56, 57].

Toll-like receptors (TLRs) detect microbial components, including glycans, to initiate immune responses [58]. They are expressed on all cells of the innate immune system and most cells of the adaptive immune system. Structurally, TLRs are type I transmembrane proteins that have leucine-rich repeats in their ectodomain for recognition of pathogen-associated molecular patterns or PAMPs [59]. The intracellular domains are composed of Toll-interleukin 1 (IL-1) receptor (TIR) domains that perform downstream signaling via crosstalk with the IL-1 pathway. Scientists have discovered around 10 functional TLRs in humans that have differential expression patterns and are specific to different ligands while also inducing different target genes [60]. The expression of TLRs is not restricted to immune cells such as macrophages, dendritic cells and lymphocytes. Across various cancers, their expression is extended to the tumor cells as well, indicating that TLRs exhibit a unique but multifaceted role in cancer progression and therapy [61–63]. Due to their crucial role in engaging both, innate and adaptive immune systems, TLRs are one of the most useful handles of cancer immunotherapy. TLR7 and TLR8 were recognized as attractive targets owing to their ability to act as an indicator of a more “immune-active” or “hot” tumor microenvironment [64]. While specific TLR7/8 agonists have been reported in the literature and have also entered clinical trials [65], the approach to conjugate them to sugar moieties for tumor targeting is unique [66]. Traditionally, TLR agonists were also conjugated to TACAs leading to enhancement in antigen presentation, dendritic cell maturation, and T-cell activation [67, 68].

As schematically summarized in Fig. 1b, the immunomodulatory impact of CLR and TLR engagement via glycoantigens can be used to generate sugar-based immunotherapeutics [69]. Interestingly, the immune system interprets the “threat level” of a TACA based on its density and chemical environment. The choice of carrier protein or a precise set of physical properties and the context of antigen presentation are required to convert a naturally weak immunogenic TACA into a vaccine (generating immunologic memory). The choice of adjuvants is one of the most important factors in determining whether the vaccine will invoke a cellular or a humoral immune response [70, 71]. Traditionally used adjuvants like alum are predominantly Th2-skewed i.e. primarily drive a humoral response [72]. The ability to mount an effective cytotoxic T-cell response is present in the Th1-dominant adjuvants such as TLR agonists (MPLA and CpG ODN) and Saponins (e.g. QS-21) [73]. These are able to ensure the secretion of Interferon-gamma (IFNγ) and the activation of cytotoxic CD8^+^ T lymphocytes (CTLs), while avoiding the suppressive Th2 skew [74]. Recognizing that single pathways are easily suppressed by the tumor microenvironment, modern oncology relies on combination adjuvant systems. Some of these including AS01 (QS-21 + MPLA) and Emulsions (e.g., MF59, Montanide ISA 51) exert a synergistic effect, thereby yielding a highly robust Th1 response and a strong immunological memory [75].

(iii) **Immunomodulation via Siglecs**: Siglecs are a family of transmembrane receptors that recognize sialylated glycans on cell surfaces [76]. These are predominantly expressed on immune cells, including natural killer cells, subsets of T cells and B cells, macrophages and dendritic cells [77–80]. Siglecs play a crucial role in regulating immune responses, maintaining self-tolerance, and modulating inflammation [81]. There are 15 different Siglecs and most of them possess cytosolic ITIMs, which upon activation (sialoglycan binding), recruit SHP phosphatases and transmit inhibitory signals to suppress immune cell activity. The expression of Siglecs on tumor associated immune cells is an additional route of modulating immune cell signaling, helping tumors evade immune surveillance [82]. Tumors exploit Siglec signaling by overexpressing sialylated glycans, effectively suppressing anti-tumor immune responses [83, 84]. Siglec-15 is unique among all Siglecs, because it exhibits a high homology with B7 family members by displaying only one IgV and one IgC2 domain [85]. Some tumors express Siglec-15 directly, example Non-Small Cell Lung Cancer (NSCLC), breast cancer, colorectal cancer, glioma and Nasopharyngeal Carcinoma (NPC) [86, 87], where they enable T-cell suppression and immune escape. Researchers have observed that the frequency of occurrence of Siglec-15 expression in tumor cells (48.3%) and tumor-associated stromal cells (33.4%), was more than the PD-L1 expression in tumor cells [86]. By exhibiting a rare expression in normal (non-cancer) cells and expression patterns that are mutually exclusive of PD-L1 on cancer cells, Siglec-15 emerges as a novel immune evasion target for glycan-based therapies. Since Siglec-15 promotes an immunosuppressive tumor microenvironment (TME), it is being explored as a novel immune checkpoint target in cancer therapy [88]. A strategy to revive antitumor immunity is to deliver immunostimulants using sialic acid conjugation to perturb the Siglec-sialic acid binding. For example, when TLR agonists like Arctigenin are delivered via sialic acid decorated carriers (Fig. 1c), the TLR6-NOX2-MAPK axis seems to activate the macrophages and restore the suppressed anti-tumor immunity [89].
(iv) ** iNKT cell activation**: It is observed that invariant Natural Killer T-cells (iNKT-cells) have a unique recognition for glycolipid antigens presented by CD1d which is a non-polymorphic MHCI-like molecule [90]. The identification of CD1d as a master immunomodulatory receptor represents a paradigm shift in immunology, transitioning from a protein-centric view of antigen recognition to one that gives the cellular lipidome its due credit. The CD1 proteins were originally identified in the late 1970 s and early 1980 s as thymocyte differentiation antigens [91]. Rigorous studies by Milstein and coworkers in the mid-1980s led to a clear understanding of the isoforms of human CD1 locus [92, 93]. Out of the five isoforms, CD1d emerged as an important receptor implicated in immunomodulation due to its ability to present lipid antigens on the cell surface [94]. The absence of polymorphism of CD1d pointed to its ability to recognize lipid structures that were either conserved in many pathogens or were essential self-lipids, functioning more like a pattern-recognition receptor (PRR) than a conventional antigen-presenting molecule. The identification of CD1d led directly to the characterization of the T-cell populations it restricts. These are collectively known as Natural Killer T (NKT) cells, so named because they often co-express the T-cell receptor (TCR) and markers typical of natural killer (NK) cells, such as NK1.1 (CD161) [95]. NKT cells are divided into two main categories based on their TCR usage and ligand specificity: iNKT cells and diverse NKT cells. It is the iNKT cells whose TCRs recognize CD1d and exist in an “effector-primed” state. The significantly compromised number and function of iNKT cells in patients with advanced cancer were attributed to a concurrent downregulation of CD1d [96]. Although CD1d as a protein was cloned and purified in mid-1980s, it was not until the mid-1990s that the “orphan” receptor was matched to its ligand [97]. Originally isolated from the marine sponge *Agelas mauritianus* as part of a screening for anti-tumor compounds, α-GalCer was discovered to be a potent activator of iNKT cells [98]. As depicted in Fig. 1d, the use of glycoconjugates like α-Galactosylceramide (α-GalCer) is known to specifically get presented by CD1d and expose the galactose for recognition by the TCR of iNKT cells [98]. This leads to iNKT cell activation and enhanced antitumor immunity via direct cytotoxicity and immune orchestration [99, 100].
(v) **Influence on Selectin-mediated immune cell and cancer cell trafficking**: Immune cell migration and cancer cell migration through vasculature is largely regulated by selectins on the surface of endothelial cells [101]. Selectins were broadly recognized as cell adhesion molecules with a central role in cell trafficking. However, in the last decade it has been established that selectins and their ligands are actively engaged in modulating immune cell function and contribute to signaling relevant to anti-tumor immunity [102]. Three types of selectins have been identified and studied over the decades:

E-selectin (CD62E), whose primary function is to mediate the adhesion and extravasation of immune cells and circulating tumor cells (CTCs), binds to sialylated and fucosylated glycans (e.g., sialyl Lewis X (sLe^x^) and sialyl Lewis A (sLe^a^)) 103]. Many aggressive cancers, including breast, lung, colon, and prostate cancers, express high levels of E-selectin ligands, allowing them to interact efficiently with E-selectin on endothelial cells [104]. Some studies suggest that E-selectin expression can also be induced on tumor cells, enhancing cell-cell adhesion, immune evasion, and tumor aggressiveness [105].

L-selectin (CD62L) is expressed on leukocytes and is known for regulation of immune cell trafficking [106–109]. The rapid shedding of L-selectin from activated leukocytes, also plays an important role in cancer [102]. L-selectin’s involvement in cancer immunotherapy offers intriguing possibilities, especially because of its contradictory roles in cancer, influencing both tumor progression and anti-tumor immunity [110]. On the one hand, enhanced L-selectin expression on tumor-specific T cells has been exploited to boost the efficacy of adoptive cell therapy models [111]. On the other hand, strategies to block L-selectins have been explored as avenues to curb metastasis [112]. Hence, the dual roles of L-selectins call for a careful and strategic therapeutic proposal.

P-selectin (CD62P) is expressed on platelets, endothelium and some tumor cells [113–116]. It promotes platelet-tumor interactions, shielding cancer cells from immune attack [117, 118]. Studies have uncovered a broad spectrum of cell types that express P-selectin and imply potential roles in tumor metastasis [103]. P-Selectin Glycoprotein Ligand-1 (PSGL-1), a heavily glycosylated protein expressed on leukocytes, primarily binds with P-selectin and enables leukocyte rolling [119]. Additionally, PSGL-1 functions as an immune checkpoint, known to inhibit the anti-tumor immunity by driving T-cell exhaustion and is therefore considered as an immunotherapeutic target [120]. Lewis antigen mimics as vaccines are shown to inhibit the interaction of selectins with their corresponding ligands. For example (as depicted in Fig. 1e), Lewis antigen conjugates inhibit binding of selectins to both, PSGL-1 on immune cells as well as Lewis antigen expressing glycoproteins like Mucins on cancer cells [121]. This branch of cancer immunotherapy lends control on cancer cell extravasation and metastasis.

The five mechanisms elucidated above guide the use of TACA-based glycoconjugates for cancer immunotherapy. Another approach to target cancer by glycoconjugates is using monosaccharides and polysaccharides having specific recognition by distinct carbohydrate-binding membrane proteins, which are overexpressed on cancer cells. The current review separately details developments in these two branches: TACA-based immunotherapeutic glycoconjugates and monosaccharide/polysaccharide-based immunotherapeutic glycoconjugates. It summarizes various categories of glycoconjugates that have been designed over the years with a primary focus on the most notable developments in the last 5 years. Each section concludes with a perspective of the trend and the mechanism of immune response (Fig. 1a-e) elicited by the different categories of glycoconjugates.

An overarching yet classified report like this will help researchers in identifying the promising approaches to explore while using glycans and their conjugates for cancer immunotherapy. Since this review is focused solely on the glycans and glycoconjugates that induce an immunotherapeutic response, therefore we have not included non-carbohydrate-based therapeutic materials and other cancer therapeutic modalities.

**Fig. 2:**
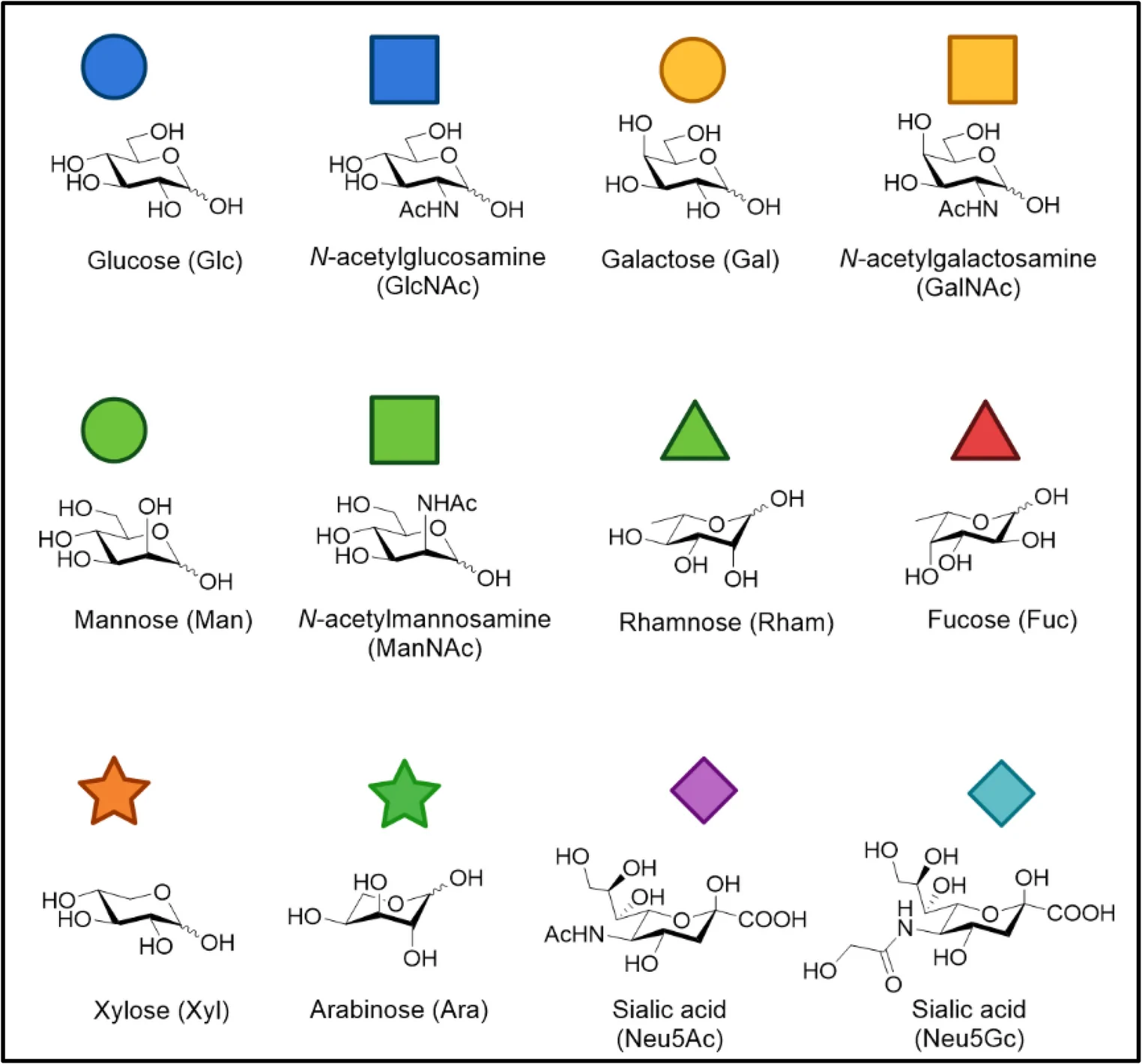
Key to representative symbols used for each monosaccharides in the figures of this review. The symbols are obtained from Biorender.com and used for generating the glycan chains represented in this review. The sugar structures are drawn using ChemDraw Professional 23

A key to representative symbols used for each monosaccharide is described in Fig. 2. Each subsection includes a figure, highlighting the different categories of glycoconjugates reported for the respective TACA, monosaccharide, and polysaccharide. The primary immune response initiated by each glycoconjugate is depicted alongside. The color scheme representing the class of these conjugates, is as follows. Orange: Small molecule; Pink: Vaccine; Purple: Nanoparticles; Green: Antibody conjugate. Figures describing TACA based vaccines also include a generic schematic of the vaccine architecture depicting the point of attachment of a chemical linker on the glycan chain. It must be understood that there is multivalent presentation of glycans on a macromolecular carrier assembly (a protein/dendrimer/liposome) and the exact number of glycan units per carrier unit is variable across systems, depending on the chemical nature of the linker and carrier as well as linkage chemistry used.

## TACA-based immunotherapeutic glycoconjugates

### TACAs on glycolipids

####  Gangliosides

Gangliosides are a distinct class of glycosphingolipids that contain ceramide lipid backbone attached to a glycan terminating in one or more sialic acid residues [122, 123]. Gangliosides are ubiquitously distributed in tissues and body fluids but are predominantly abundant in the plasma membrane of neural tissues, where they are primarily involved in cell signaling, adhesion, and modulation of receptor activity [122, 124, 125]. Structurally, gangliosides are classified according to the number of sialic acid residues: e.g., GM1, GD1a, GT1b (mono-, di-, and tri-sialylated species, respectively) [126, 127]. Specifically, GD2 and GM2 are overexpressed on the surface of neuroblastoma, melanoma, sarcomas and small cell lung cancer cells [11, 14, 128]. These gangliosides play crucial roles in tumor progression, metastasis, and immune evasion [129]. Gangliosides are known to modulate immune responses by establishing an immunosuppressive tumor microenvironment [130]. Shedding of gangliosides from tumor cells can inhibit dendritic cell activation and natural killer (NK) cell function, facilitating tumor immune evasion [131–135]. All these traits together make gangliosides potential targets for immunotherapy [129]. Initial studies in the field revolved around antibody developments, which led to notable preclinical success [136]. Anti-GD2 monoclonal antibodies like dinutuximab, have recently been developed and gained FDA-approval for treatment of high-risk neuroblastoma [137, 138]. Apart from monoclonal antibodies, gangliosides are also being studied for vaccine development, and Chimeric Antigen Receptor (CAR) T-cell engineering [139–141]. Initial attempts of ganglioside-based vaccines showed success at the early clinical stages, especially for advanced breast cancer and melanoma [142, 143]. Following the promising results of ganglioside-based vaccines, researchers explored various other multivalent scaffolds such as calixarenes and carbon-based nanomaterials to enhance the immunogenic properties of ganglioside mimetics [144–146]. A bivalent vaccine comprising GM2- and GD2-KLH, elicited a highly effective immune response [147]. In another study, immunization with a combinatorial vaccine, prepared by conjugating GD2 and GD3 with carrier protein KLH, has been demonstrated to elicit a strong immune response when co-administered with a glycan adjuvant, β-glucan [148]. In phase II clinical trials, the enhanced overall survival of neuroblastoma patients receiving this formulation, was attributed to strong and robust antibody production [149].

**Fig. 3:**
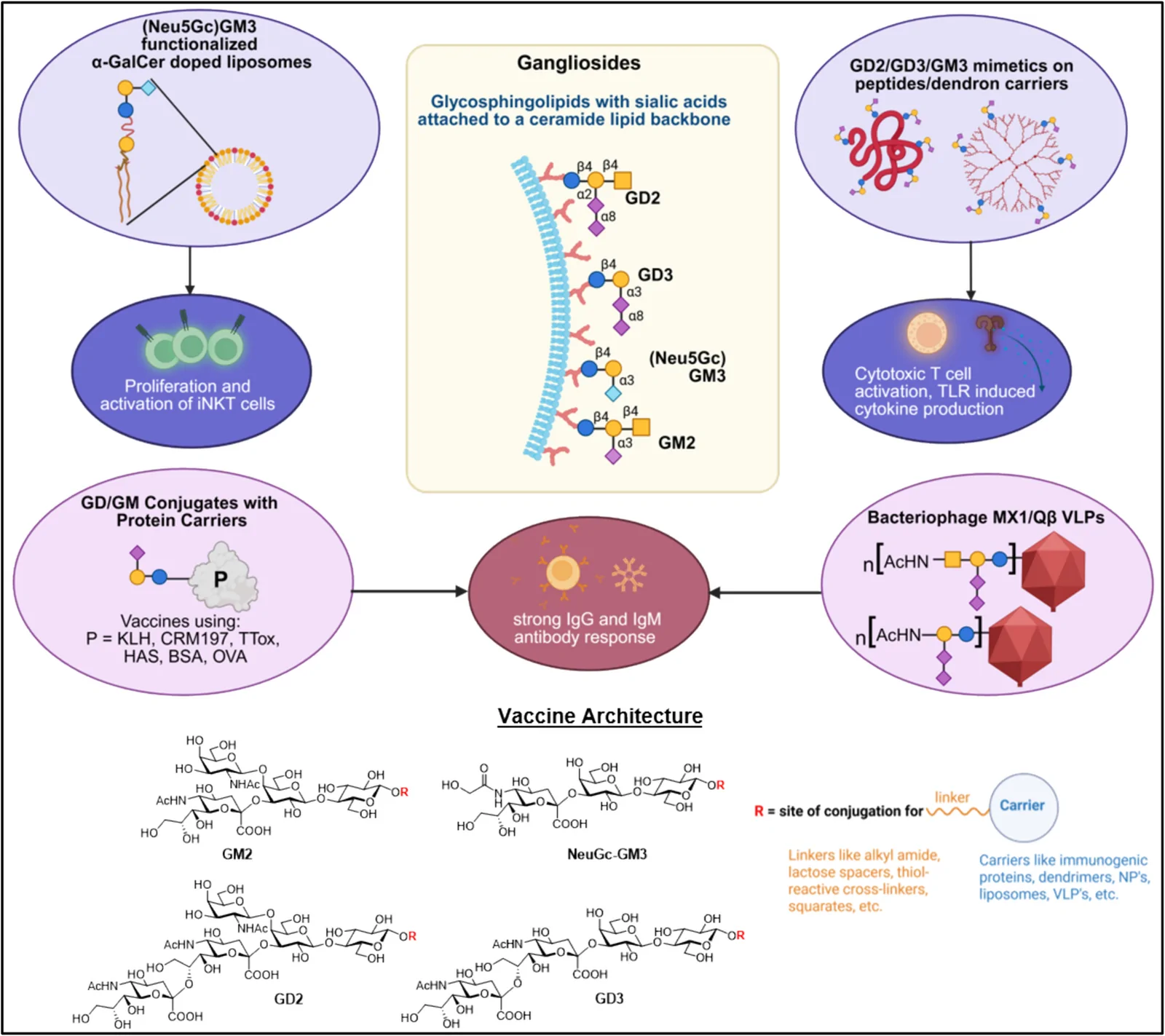
Representative categories of ganglioside-based glycoconjugates proposed for cancer immunotherapy. Ganglioside mimics have been anchored on different protein carriers and viral particles to obtain strong IgG responses. Multivalent demonstrations on peptide assemblies, dendrons and liposomes has enhanced activation of cytotoxic T cells

Soon after, Wu et al. demonstrated that a chemically stable GD2 mimic, 9NHAc-GD2 is a strongly immunogenic vaccine candidate in conjugation with bacteriophage Qβ virus-like particle. Serological analysis after immunization in non-tumor bearing C57BL/6 mice and beagles (canines) where this candidate was able to overcome the problem of hydrolytic instability, and the consequent loss in clinical efficacy of the more immunogenic form of GD2 antigen i.e. 9-*O*-acetyl-GD2 [150]. Researchers have also utilized multivalent polyamidoamine scaffold (PAMAM) to attach gangliosides namely GD2 and GD3. PAMAM-GD2 and PAMAM-GD3 vaccines prompted robust selective humoral and cellular immunity, wherein rapid expansion of CD8^+^ and δγ T cells were observed in lymphoma and melanoma models (EL4 and B16F10 cells) in C57BL/6 mice [151]. The use of OPT-821, a synthetic/highly purified fraction of QS-21, as the primary adjuvant has been instrumental in advancing several multivalent ganglioside vaccines to Phase II and III clinical trials [152, 153]. Recently, Rosenbaum et al. developed a trivalent vaccine using a combination of GM2-, GD3L-, and GD2L-KLH with OPT-821 [154]. It is evident that over the years scientists have tried to anchor GD/GM glycans on protein carriers, immunogenic liposomes, amphiphilic peptides and dendrimer-based carriers [18, 155–158]. The last decade has seen a vast surge in the development of ganglioside-directed vaccines with ganglioside antigens with or without other TACAs [129, 141]. The notable examples are categorized and summarized in Fig. 3.

Very recently, Zhao et al. reported the construction of bacteriophage MX1 virus-like particles (VLPs) functionalized with ganglioside antigens as an effective cancer immunotherapy strategy [159]. The study described the synthesis of isothiocyanate derivatives of NHAcGD2 and NHAcGD3 via chemoenzymatic routes. Further, these derivatives were conjugated with the amine group of purified MX1 VLPs utilizing thiourea linkage chemistry to produce MX1-NHAcGD2 and MX1-NHAcGD3 conjugates. Immunization with MX1-NHAcGD2 and MX1-NHAcGD3 conjugates in non-tumor bearing C57BL/6 mice showed the induction of high IgG titers, suggesting a strong immunogenicity. Additionally, antibodies produced from MX1-NHAcGD2 and MX1-NHAcGD3 fabricated glycovaccines displayed high binding specificity towards IMR-32 neuroblastoma cells and SK-MEL-28 melanoma cells, respectively. Furthermore, it was also shown that sera extracted from vaccinated mice effectively killed the respective tumor cells through complement-mediated cytotoxicity. The authors concluded that these two vaccines have the potential to target cancers expressing GD2 and GD3 antigens at the clinical level.

In parallel, Romanò et al. developed ganglioside-functionalized nanoparticles using GM3 and (Neu5Gc)GM3 [160]. After a stereoselective synthesis of the ganglioside antigens, the authors conjugated those to linkers having a reactive NHS ester that was then covalently conjugated to α-Galactosylceramide (αGalCer). Stable liposomal nanoparticle formulations were prepared using distearoylphosphatidylcholine (DSPC) and cholesterol mixed with these glycolipid-TACA conjugates followed by lipid extrusion to achieve uniform size < 200 nm. Since αGalCer is a known agonist of iNKT cells, the authors performed co-culture experiments using human dendritic cell (DC) and iNKT cells. Interestingly, both the liposomal formulations i.e. those containing GM3-αGalCer and those containing its combination with (Neu5Gc)GM3-αGalCer showed improved presentation of CD1d on human DCs while also significantly promoting proliferation and activation of human iNKT cells. In a GM3-expressing B16F10-based melanoma mice model, the vaccinated mice showed robust IgG responses, CDC-mediated tumor regression and overall, an effective anti-tumor immune response in the absence of any external adjuvant. As evident from Table 1, gangliosides have been thoroughly investigated for vaccine development over a couple of decades now. However, limitations of antigen shedding and potential off-tumor toxicity continue to fuel the requirement for more strategic approaches [161].

#### Globosides

Globosides constitute a family of neutral glycosphingolipids that are characterized by their carbohydrate headgroups comprising galactose and *N*-acetylgalactosamine units [162]. These glycolipids are decorated at the outer leaflet of the plasma membrane and are involved in cell-cell recognition, adhesion, and extracellular signaling [163, 164]. Specific globosides like Gb3 and Globo H are aberrantly overexpressed on many types of tumor cells, such as in breast, colon, ovarian, and prostate cancers. Particularly, Globo H is overexpressed in most epithelial tumors and has been recognized as one of the many tumor-associated carbohydrate antigens (TACA) that are suitable for designing targeted therapy [165]. In recent decades, scientists’ interest has significantly grown to develop a large number of globoside-based vaccines. At first, research in this field has seen an increased number of publications reporting the use of *E. coli* for expression of glycotransferases that gave high yields of the enzyme ensuring large-scale synthesis of globopentaose (Gb5), Globo H, and SSEA-4 [166]. The chemoenzymatic synthetic approaches have also been coupled with efficient sugar nucleotide regeneration systems. Wong, Danishefsky and coworkers have reviewed the details of preparation of various Globo series vaccines and their therapeutic potential [167]. However, these cancer vaccines have faced challenges with regard to consistently eliciting strong T-cell mediated immunity [168].

To address these challenges, researchers started exploring fully synthetic glycoconjugate vaccines by incorporating immune-stimulating adjuvants to enhance both humoral and cellular immune responses [169]. Some of the early reports demonstrated the total synthesis of the Globo H glycosphingolipid [170]. Subsequently, researchers prepared synthetic vaccines by conjugating this presynthesized Globo H (GH) with a carrier protein KLH. When these synthetic vaccines were used in a murine breast cancer model, in combination with adjuvants QS-21 and Bovine Serum Albumin (BSA), the authors observed the production of specific antibodies against breast cancer cells, MCF7 [171]. Later, GH-KLH vaccines along with adjuvant QS-21 were tested in human patients and found to elicit immunological responses [172–174]. To elaborate further on the similar rationale, various carrier proteins including KLH, and diphtheria toxoid (CRM197), were conjugated to GH [175]. The resulting conjugates along with a synthetic adjuvant α-galactosylceramide (C34) elicited strong and robust antibody production. Notably, GH-DT/C34 vaccine prompted more IgG antibodies compared to a phase III clinical vaccine GH–KLH/QS21, with enhanced specificity towards breast cancer cells expressing GH, SSEA3 and SSEA4. Further, these findings led to the development of another effective vaccine candidate, SSEA4-DT/C34, which upon immunization produced highly specific antibodies against SSEA4. A mechanistic study unraveled the role of β1,3-galactosyltransferase V (β3GalT5) enzyme in tumor progression and metastasis in cancer cells expressing Globo series glycosphingolipids (GSLs) [176]. The findings established a correlation between Globo series GSLs (SSEA3/SSEA4/Globo-H) and the FAK/CAV1/AKT/RIP complex in the tumor, demonstrating an effective therapeutic strategy based on the synergistic effect of anti-Globo-H and anti-SSEA4 monoclonal antibodies.

Recently, Wong and coworkers uncovered the mechanistic action of Globo H-DT/C-34 vaccine by systematically studying its mechanism of action [177]. The authors discovered that Globo H-DT/C-34 conjugate is taken up by DC’s, enzymatically processed and subsequently, the resulting glycans are presented by MHC II molecules that are recognized by B-cells to produce specific antibodies against the GH and SSEA3 glycans. The study also discussed the activation of CD4^+^ cells through MHC-II antigen complexation of dendritic cells. In addition, this study clearly demonstrated that FUCA1 present in dendritic cells enzymatically removed the terminal fucose from GH-DT vaccine to produce SSEA-3 and modified glycans for presentation. Further, the authors improved the conversion of Gb4 to Gb5 (SSEA-3) glycan by employing insect cell purified galactosyl transferase, β3GalT5-1. The previously known microbial glucotransferases LgtD were recently used for the conversion of Gb4 to Gb5 despite moderate yield [178]. This study demonstrated the development of an effective enzyme S66A β3GalT5-1 variant by identifying two isozymes of human β3GalT5, crucial for producing GH series glycans in cancer cells, followed by alanine-scanning mutagenesis. The newly developed glycoform, S66A β3GalT5-1 expressed and purified from Sf9 cells displayed 10-fold greater efficiency than LgtD for the production for GH series glycans. In a unique approach, Ghosh et al. conjugated Globo H hexasaccharide to the zwitterionic polysaccharide PS-A1 via a stable oxime linkage [179]. When tested in healthy C57BL/6 mice with adjuvants, the “entirely carbohydrate-based” conjugate elicited a robust, antigen-specific immune response in the form of high titers of highly specific IgG and IgM antibodies. These antibodies were able to bind to Globo H-positive cancer cells in culture (MCF-7 and OVCAR-3) and successfully mediate complement-dependent cellular cytotoxicity.

**Fig. 4:**
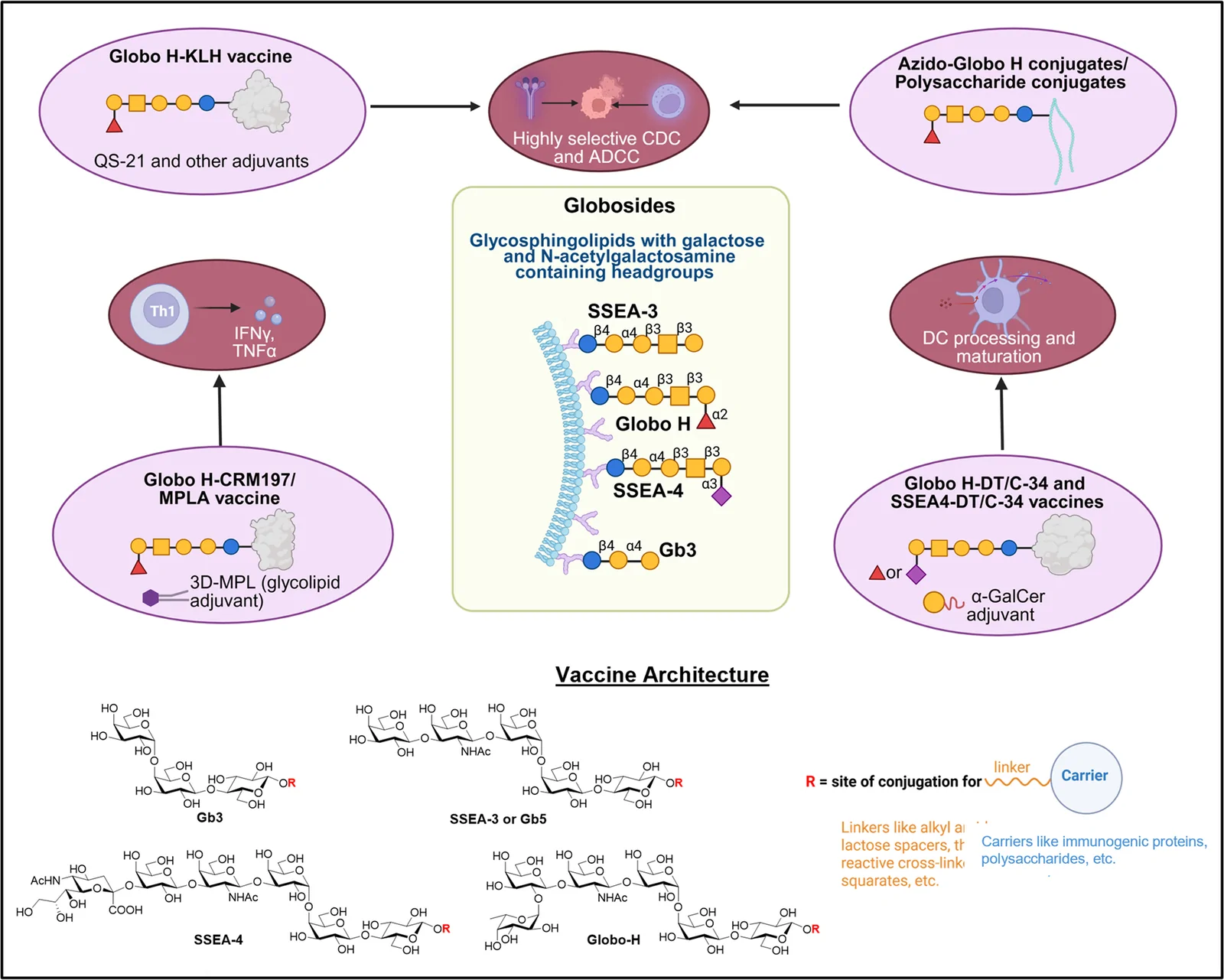
Representative classes of Globoside-based cancer vaccines. Globo-H antigens have been conjugated to various protein carriers and formulated with different adjuvants to achieve Globo-H selective ADCC, helper T cell activation and DC maturation. Zwitterionic polysaccharides functionalized with azido-Globo H units, have shown significant promise in eliciting antigen-specific tumor cell killing

Vaccines targeting Globo H, such as Adagloxad Simolenin (OBI-822), conjugate the Globo H hexasaccharide with KLH to induce an immune response against Globo H-expressing tumors [180]. A placebo-controlled Phase II study (NCT01516307) in patients with metastatic breast cancer (MBC) revealed a strong correlation between humoral immune response and clinical outcome. This finding was instrumental in the field, as it suggested that the efficacy of the vaccine was contingent upon the quality of the patient’s immune response, leading the way for personalized clinical designs. Moving forward, Globo series vaccines, along with saponin-based adjuvants, have been evaluated for safety and efficacy in more defined populations such as patients with early stage disease [181, 182]. This formulation (OBI-822) was then pushed for a Phase III clinical trial, GLORIA, in patients with high-risk triple-negative breast cancer (TNBC) in the adjuvant setting [183]. Unlike the Phase II study, the GLORIA trial integrated a mandatory IHC screening to select patients with appropriate Globo H expression levels. Recently, Ma et al. reported that chemoenzymatically derived Globo H-CRM197 conjugate along with a combination of adjuvants QS-21 and 3D-MP, prompted a robust immunological response [184]. In this report, the authors discussed a streamlined chemoenzymatic synthesis of Globo H antigen and its conjugation to a carrier protein, CRM197 (a non-toxic form of diphtheria toxin). Additionally, the study discussed an efficient synthesis of 3-O deacyl-monophosphoryl lipid A (3D-MPL) as an adjuvant. Formulation of Globo H-CRM197 with different adjuvants such as QS-21, 3D-MPL or S34 (separately or in combination) showed an antibody response and Th1 cellular immunity in Balb/c mice, with the combination of 3D-MPL and S34 showing the strongest response. The IgG antibodies extracted from immunized mice exhibited specific binding affinity for Globo H-positive cells along with enhanced complement-dependent cytotoxic and antibody-dependent cellular phagocytic activity. Despite showing strong antitumor activity in vitro, the vaccination did not prompt antitumor activity in 4T1 tumor bearing Balb/c mice. The progress in this field has been rather rapid over time and the different vaccine classes developed so far have summarized in Fig. 4. The most notable examples of Globo H based vaccines are summarized in Table 1. Earlier this year, the pivotal GLORIA study (NCT03562637) was terminated due to safety concerns and lack of likelihood of meeting its primary efficacy endpoint [185]. This termination has prompted an immediate redistribution of resources and a generic industrial pivot towards alternative technologies such as ADCs and bispecifics. The development of Globo H targeted antibodies and CAR T-cells is also ongoing, expanding the realm of directed cancer immunotherapy.

### TACAs on glycoproteins

#### Lewis antigens

Lewis antigens are a family of carbohydrate structures, including Lewis^x^ (Le^x^), Lewis^y^ (Le^y^), sialyl-Lewis^a^ (sLe^a^), and sialyl-Lewis^x^ (sLe^x^), whose expression is essential during embryonic development and decreases significantly in adult tissues [186–188]. Cancerous cells show an aberrantly upregulated expression of these antigens in their glycocalyx and that has been discovered to contribute to tumor progression, metastasis, and immune evasion [106, 189–191]. Sialyl-Lewis^a^ and sialyl-Lewis^x^ exhibit strong binding affinities for E-selectin, P-selectin, and L-selectin, the adhesion molecules that are expressed on endothelial cells and leukocytes [102, 192, 193]. By virtue of the overexpression of Lewis antigens, tumor cells exploit E-selectin-mediated adhesion to vascular endothelium, enabling them to arrest in distant organs and extravasate into tissues, facilitating metastatic spread [194].

Due to their cancer-associated expression, Lewis antigens are attractive targets for immunotherapeutic approaches, including vaccines, monoclonal antibodies, and glycan-engineered CAR T cells [41, 168, 195, 196]. A handful of Lewis antigen- based cancer vaccines have made their way to clinical trials and are being constantly improved [164, 197]. Being relatively small, Lewis antigens are considered weak immunogens as they may often fail to induce robust adaptive immune responses unless conjugated to carrier proteins. To enhance their immunogenicity, researchers have conjugated Lewis antigens to carrier proteins such as keyhole limpet hemocyanin (KLH) or diphtheria toxoid (CRM197) and observed the stimulation of robust immune responses, particularly in Balb/c mice model [198, 199] Serological analysis post-immunization in non-tumor bearing mice was the primary method of assessment of antibody titer in these reports. However, the report by Kawamura et al. also showed that once the mice were immunized SSEA1-CRM-197 conjugate, then the transplantation of iPSC-CMs (induced pluripotent stem cell-derived cardiomyocytes) led to engraftment without the otherwise observed tumor formation. Lewis antigen sLe^a^ was also shown to elicit high IgG titers when conjugated with KLH and administered with adjuvants such as GPI-0100 or QS-21 in non-tumor bearing C57BL/6 mice model [200]. The generated IgG and IgM antibodies were shown to specifically recognize sLe^a^-positive cancer cells (SW626 cell line) and induced complement-dependent cytotoxicity (CDC) in vitro, establishing the vital roles of carrier protein and the adjuvant in prompting an effective immune response. Diab et al. demonstrated that sLe^a^-KLH vaccine, together with the immunologic adjuvant QS-21, enabled the generation of strong antibody responses against sLe^a^ upon subcutaneous injection in patients [201]. Interestingly, a highly specific and strong-binding antibody for sLe^a^ has been isolated from the sera of patients receiving sLe^a^-KLH vaccine and named MVT-5873 [202]. This unique glycan-induced immunotherapy by Gupta et al. has shown tremendous promise and has made its way to clinical trials for pancreatic cancer. This antibody demonstrated exquisite specificity to sLe^a^ antigen with no cross-reactivity to other related antigens like Le^a^, sLe^x^, or Le^y^. These clinical trials are currently ongoing with patients having metastatic pancreatic cancer and elevated levels of Carbohydrate Antigen 19 − 9 (CA 19 − 9) in the blood. Subsequent studies showed that antibodies derived from human patients immunized with sLe^a^-KLH vaccine was not impressive in preclinical studies. Further to achieve enhanced therapeutic effect, the Fc portion of the antibodies was re-engineered [203].

The last five years have seen parallel advancement in both the fields, synthetic strategies for Lewis antigen based glycoconjugates as well as innovations in glycoconjugate vaccine platforms/carrier systems. Recent research by Guo and coworkers has highlighted the efficacy of one-pot preactivation and iterative glycosylation for synthesis of complex Lewis-antigens [204]. This method involves the sequential activation of thioglycoside donors leading to fast building of hexasaccharide and nonasaccharide cores without the need for purification at each intermediate step. This study also highlighted the use of specific protecting groups, such as *N*-phthalimido group for glucosamine and benzyl or benzoyl groups for galactose, is important to maintain regiospecificity and stereoselectivity of the β−1◊4 and α−1◊2 linkages. In a much newer synthetic strategy, scientists used Umemoto’s reagent and Cu(OTf)_2_ under UV irradiation for the synthesis of complex KH-1 antigen which needed the glycosylation of sterically hindered acceptors [205].

Regarding recent developments in antigen presentation formats for efficient induction of a class switch to high-affinity IgG antibodies, Synthetic Lewis antigens (Le^x^, Le^y^, KH-1) linked to KLH via a lactose spacer, have been demonstrated as effective immune sensitizers for Le^x^/Le^y^ rich tumor cells [206]. High-throughput studies have been performed to establish comparative structure-immunogenicity relationships of the various Lewis antigens. A crucial insight from this research is that the native spatial presentation of antigens in the case of a synthetic glycoconjugate may be different from that on the tumor cell surface. As a result, linker length and density need to be carefully optimized to match the natural tumor glycocalyx. This research has also led to the development of one of the most impactful strategies to enhance the immunogenicity of Lewis antigens, i.e. fluorination of the carbohydrate structure. It leads to the creation of “non-self” mimics that can break central tolerance (instigate an immune response) while still maintaining the conformational integrity required for cross-reactivity with the native antigen.

**Fig. 5:**
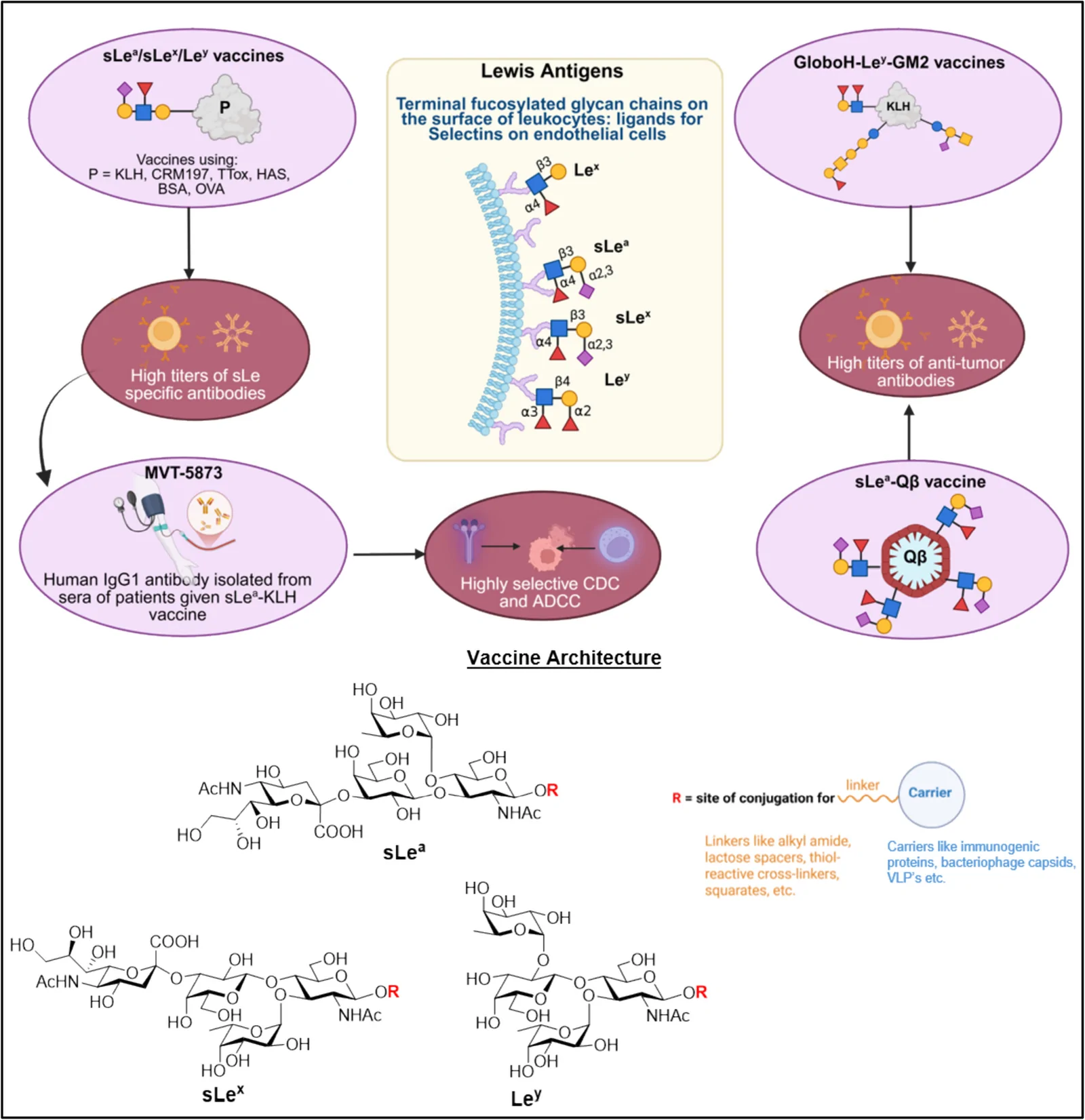
Lewis antigen-based cancer immunotherapy. Lewis antigen mimics have been conjugated to various protein carriers to enhance immunogenicity and antigen-specific humoral responses. A notable example is of sLe^a^ glycan chains conjugated to bacteriophage capsid Qβ

As opposed to conventional protein-based carriers, Rashidijahanabad et al. prepared a Sialyl Lewis^a^ (sLe^a^) functionalized bacteriophage Qβ conjugate and observed a strong and robust anti-sLe^a^ IgG antibody response [207]. The study described a stereoselective synthetic route to synthesized sLe^a^ tetrasaccharide antigen which was later conjugated onto bacteriophage Qβ particles to produce stable Qβ-sLe^a^ conjugates. Upon immunization in C57BL/6 mice, Qβ-sLe^a^ conjugate induced strong and long-lasting anti-sLe^a^ IgG antibodies at levels orders of magnitude higher than that of KLH-sLe^a^ vaccine. The authors demonstrated that extracted anti-sLe^a^ antibodies were highly selective towards sLe^a^ epitopes expressed on engineered mouse ductal adenocarcinoma cells (FC1199FB) and induced apoptosis through complement-mediated cytotoxicity. Mice immunized with Qβ-sLe^a^ conjugates, after challenging with sLe^a^-positive tumor cells showed high anti-sLe^a^ IgG titers and significantly reduced tumor development in a metastatic murine melanoma model. It is evident that the field of Lewis antigen-based targeting for cancer immunotherapy has progressed significantly from basic screening and identification to diverse therapeutic strategies (Fig. 5). Promisingly, ongoing research is focused on optimizing each approach for clinical translation (Table 1).

#### Truncated mucin-type O-glycans

This group of glycans represents the “oversimplification” of the cell’s glycome i.e. they are the “starting points” of the *O*-glycosylation pathway unique to cancer cells [208]. Instead of forming long, healthy glycan chains, oncogenic transformation leads to formation of truncated glycan chains, resulting in three specific structures, Thomsen-nouveau (Tn), sialyl- Thomsen-nouveau (sTn), and Thomsen-Friedenreich (TF). These are collectively called “Mucin-type” O-glycans because they were first discovered and are most abundant on a glycoprotein called Mucin. The term “mucin-type glycosylation” refers to the process where GalNAc is attached to the oxygen atom of a Serine or Threonine amino acid [209]. This GalNAc attachment is a “signature” of how mucins are built, and any glycan chain starting in this manner is classified as “mucin-type,” even if it is found on a non-mucin protein. Mucins are heavily glycosylated, high molecular weight proteins mainly expressed on the apical surface of epithelial cells [210]. Their main function is to protect and lubricate mucosal tissues by maintaining mucosal barrier integrity and hydration. There are about 20 members of the mucin family of glycoproteins and each of them forms a filamentous “brush-like” structure bearing hundreds of glycan chains along its bristles [211]. One of the commonly observed hallmarks of cancer is the presence of aberrantly glycosylated Mucins on the cell surface [212]. For example, MUC1 is overexpressed and hypoglycosylated in many carcinomas [213]. The loss in polarized distribution of this glycoprotein leads to exposure of many of the TACAs, such as Tn, sialyl-Tn (STn), and sialyl-Lewis^a/x^ (sLe^a^/sLe^x^) [214]. These unique features of Mucins have attracted researchers to develop cancer vaccines for quite a few years [215, 216].

**Fig. 6:**
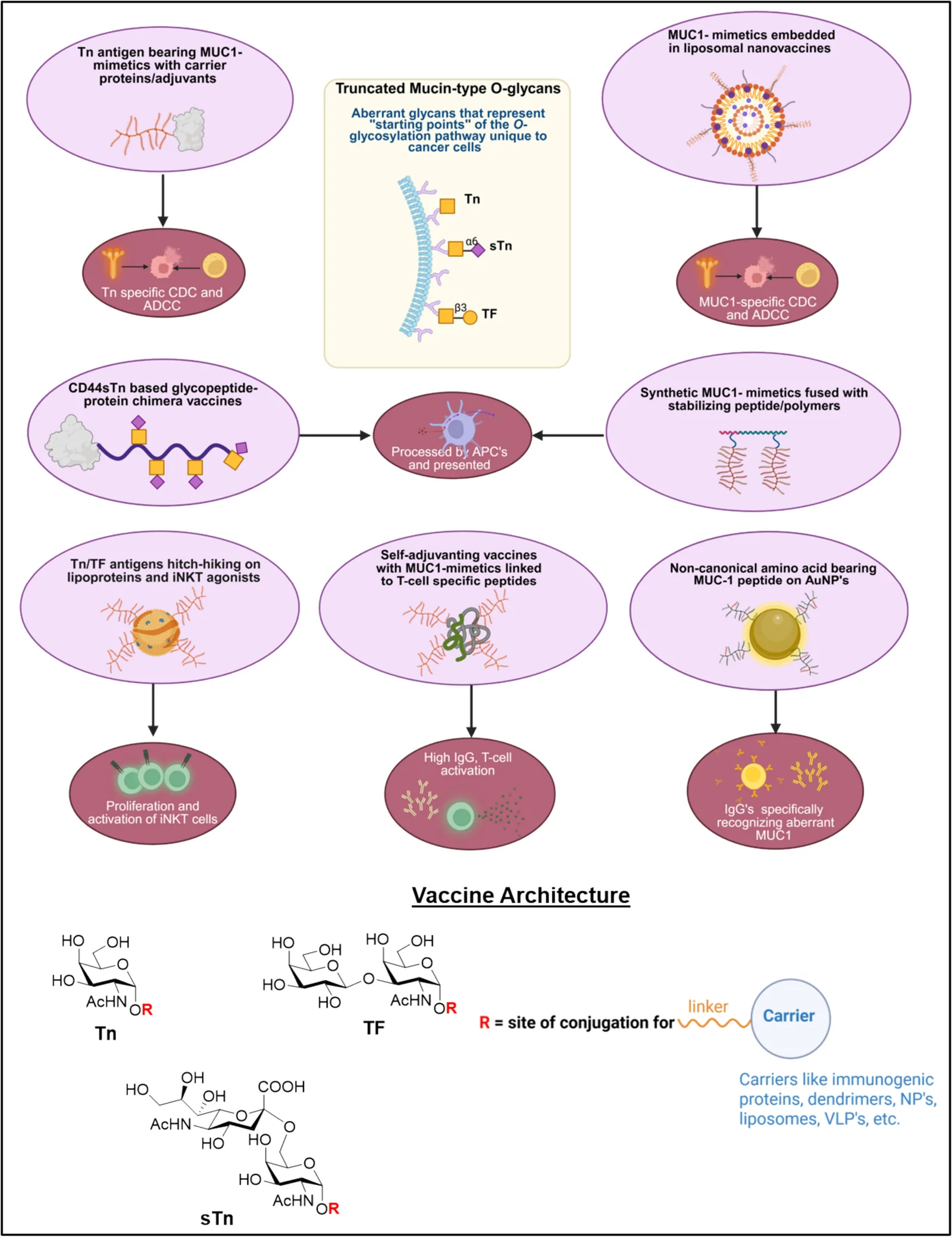
Cancer immunotherapy strategies based on truncated Mucin-type O-glycans. Antigen-bearing mucin backbones have been presented on protein carriers, liposomes, polypeptides, lipoproteins and gold nanoparticles to achieve high anti-tumor immune response. sTn bearing CD44 frameworks have also been used for generation of chimeric vaccines for DC mediated immunologic memory

One of the early attempts regarding vaccine development against MUC-associated TACAs is the pioneering work by G. F. Springer who demonstrated that the Thomsen-Friedenreich (TF) antigen in breast cancer patients elicits cell-mediated immune responses [217, 218]. These immune responses were specific to women who suffer from breast cancer and rarely immunogenic in healthy individuals. Based on these studies, researchers further developed TF (Galβ1 − 3GalNAc − α − *O* − Ser/Thr) and Tn (GalNAc − α − *O* − Ser/Thr) antigen-based diagnostic and immunotherapeutic strategies [219]. Further, Springer and coworkers developed the first murine antibodies to target Tn antigen, later Hakomori developed a monoclonal antibody (mAb) against sTn [220, 221]. Subsequently, many other antibodies were discovered against MUC-associated antigens, however, this generation of antibodies lacked specificities [222, 223] In recent decades researchers have given significant attention to develop MUC antigen-based vaccines [224–229]. One such example is Theratope or sTn-keyhole limpet hemocyanin conjugate vaccine, that underwent clinical trials for breast cancer but exhibited limited efficacy [230–232]. The setback was attributed to patients’ selection with tumor having lower sTn expression. Further studies included the use of peptides to develop vaccines [229, 233–235]. For example, MUC1 lipopeptide vaccine, L-BLP25 showed excellent results in Phase II trials for NSCLC although the Phase III trial did not establish a broader implication [235, 236]. The limited outcome from early vaccine development unraveled the intricacies of TACAs and tumor microenvironment and potentiated the move towards customized immunotherapies. Recent refinements in the development of MUC antigen conjugated vaccines included MUC1 peptide backbones, T-helper epitopes and potent adjuvants with TACAs [67, 164, 237–239]. As summarized in Fig. 6, researchers have consistently been improving the vaccine candidates by incorporation of liposomes, virus-like particles and gold nanoparticles [238, 240–243]. The goal has been to enable stable transportation of the intact Mucin antigens and improve antigen presentation, thereby ensuring strong immunotherapeutic outcomes along with avoiding non-specific immune instigations [164]. Over the last three years, outstanding developments have been reported to augment Mucin-targeted cancer immunotherapy, and a few relevant developments are highlighted here.

To overcome the susceptibility to endogenous glycosidases, Wu et al. reported the synthesis and immunological evaluation of an unnatural, structurally modified β-linked MUC1 TF conjugate [244]. By strategically altering the topology of the glycosidic linkage, the authors designed a glycopeptide mimic that exhibited improved resistance against enzymatic cleavage while still maintaining the precise molecular presentation required for immune recognition. In a unique approach, Pifferi et al. have shown a strong immunogenic response in a mouse model by a synthetic tri component vaccine composed of tumor-specific MUC1 antigen, saponin-based immune-stimulating molecule and helper T-cell peptide [245]. The authors conjugated synthetic MUC1 glycopeptides to saponin scaffold followed by attachment to PV helper T-cell epitope peptide using orthogonal chemical ligation. Further, the study described that the tri-component conjugate self-assembled in aqueous solution exposing MUC1 antigens on its surface. Upon injection of the tricomponent vaccines in female C57BL/6 mice, a strong and long-lasting antigen-specific antibody response (especially IgG1 and IgG2b) was observed. Serum from tri-component vaccinated mice specifically recognized TA-MUC1 on MCF-7 cells and NMR studies demonstrated that the superior immunogenic response of tri-component vaccines compared to di-component controls was associated with aggregation behavior and stability of the tri-component vaccines. The fascinating part about this study was the induction of robust immunogenic response of the three-component vaccine without the need for external adjuvants or carrier proteins. Murphy and coworkers prepared MUC1 Tn-functionalized glycopeptides containing a synthetic *S*-GalNAc glycocluster and studied their immunomodulatory function [246]. The authors first synthesized a glycocluster with three α-*S*-GalNAc residues grafted to a tetraphenylethylene (TPE) scaffold containing an amino acid linker (TPEaa) and then they prepared several fragments of MUC1 glycopeptides with or without T helper epitope P30. The glycocluster was later fabricated into a modular vaccine construct, incorporating the MUC1 Tn glycopeptides and a P30 T-helper epitope. Exposure of MUC1 Tn fabricated glycopeptides to DC’s exhibited enhanced receptor-mediated uptake via MGL receptor. Following immunization with MUC1 Tn fabricated glycopeptide vaccines, C57BL/6J mice (adult female) produced specific antibodies against MUC1 Tn. The authors concluded that MUC1 Tn fabricated glycopeptide vaccines bearing the GalNAc glycocluster prompted a high titer of anti-Tn-MUC1 antibodies without causing loss of selectivity for the target antigen.

Recently, Jayaraman and coworkers have demonstrated that the complexation of Tn antigen glycolipid and BSA form a stable micelle that induces humoral response in lymph nodes to produce specific antibodies against Tn antigen [247]. The authors synthesized Tn antigen glycolipids by coupling the anomeric α-serine to PEG-linked phospholipid, Distearoylphosphatidylethanolamine (DSPE). This study discussed the micellization of glycolipids in PBS and demicellization in presence of BSA to form glycolipid-BSA micelle. The Tn-glycolipid-BSA complex was found to “hitch-hike” to the lymph nodes and lead to a strong immune response. When compared to a covalently conjugated complex of Tn and BSA, it can induce around 8–10-fold higher antibody titers in Balb/c mice model. Zhou et al. reported self-adjuvanted multivalent MUC1 glycopeptide vaccines that prompted a robust antitumor immune response without the need for external adjuvants [248]. Initially, MUC1 tandem repeat peptides glycosylated at T9, S15, and T16, with Tn antigens were prepared. Subsequently, MUC1 glycopeptides were conjugated with T-helper cell epitope, P30 peptide, and Pam3CSK4 (a lipopeptide that is a TLR-2 ligand) to generate mono and trivalent self-adjuvanted vaccines. When multivalent glycopeptide vaccines were injected into wild-type and MUC1 transgenic mice, the wild-type mice produced significantly higher titers of MUC1-specific antibodies belonging to subtypes IgG1, IgG2b, and IgG3, implying an effective humoral immune response. When the vaccinated mice were challenged with B16-MUC1 tumor cells, they showed delayed tumor development and prolonged survival, particularly those treated with the trivalent vaccine. These findings suggest that the self-adjuvanted multicomponent MUC1 vaccines hold potential for clinical use. This research group has further advanced the field and very recently developed a Tn-DSPE-PEG-2000 amphiphilic vaccine that is able to achieve endocytic delivery of the Tn antigen for improved immune presentation [249]. In a very creative way, they have also synthesized multivalent Tn epitopes using first-, second-, and third-generation poly(ether imine) (PETIM) dendrimers. While demonstrating a valency-dependent immune response, this study conveyed that increasing antigen density on synthetic scaffolds can overcome the low immunogenicity of TACAs [250].

Nishat et al. reported a new glycopeptide-based vaccine, Tn-PV-PS A1 that induces Tn specific cytotoxic T lymphocytes [251]. They described the synthesis of Tn-functionalized glycopeptide derivative, Tn-PV-ONH₂ which was then selectively tethered to oxidized zwitterionic polysaccharide carrier PS A1 using oxime chemistry to produce Tn-PV-PS A1 vaccine. The experimental data showed that the Tn-PV-PS A1 induced strong T-cell proliferation compared to unconjugated Tn-PV peptide. Additionally, isolated CD4^+^ T cells and CD8^+^ T cells from C57BL/6 mice treated with Tn-PV-PS A1, showed strong affinity to Tn-PV peptide. Finally, the study demonstrated that the isolated CD8^+^ T-cells secrete a significant amount of interferon gamma and it specifically recognizes murine EL4 tumor cells expressing Tn MUC1 antigen and induces apoptosis. The authors concluded that the large zwitterionic PS A1 carrier might have facilitated MHC-1 cross-presentation, which is an unusual feature for carbohydrate-based carriers. Weng et al. constructed a sialyl-Thomsen–Friedenreich (sTF) antigen using chemoenzymatic synthesis followed by conjugation with CRM197 to produce sTF-CRM197 vaccine [252]. Immunizing Balb/c mice with sTF-CRM197 conjugate with or without KRN7000 produced a significant amount of specific IgG antibodies. Further, the study described that the antisera induced by sTF-CRM197 vaccine displayed specific affinity for T-47D breast cancer cells expressing sTF antigens and induced cell killing through complement-dependent cytotoxicity in vitro. The immunized mice were investigated for 42 days where an increase in IgG titers with IgG1 predominance and a decrease in IgM titers was noticed over time, implying class switching and establishment of long term secondary immune responses.

Bermejo et al. demonstrated an effective immune response in Balb/c mice elicited by MUC1 glycopeptides, having noncanonical amino acids, fabricated on gold nanoparticles [253]. The study demonstrated the synthesis of MUC1 peptides with or without α-O-GalNAc by replacing the threonine residue at the immunodominant APDTRP epitope with noncanonical amino acids. The binding studies showed that a synthetic MUC1 glycopeptide containing the noncanonical amino acid (2 S,3R)−3-hydroxynorvaline, 1-Hnv* displayed similar or higher binding affinity to anti-MUC1 antibodies compared to natural counterparts 1-Thr*. Conformational and antibody-antigen complexation studies reveal that Hnv-containing glycopeptide mimics the natural counterparts 1-Thr* with increased CH/π interactions that resulted in strong and stable binding. Further, glycopeptide 1-Hnv* was immobilized on gold nanoparticles to evaluate immunological response in non-tumor bearing Balb/c mice. Upon immunization, the generated anti-MUC1 IgG antibodies specifically bound to MUC1-positive breast cancer T47D cells over MUC1-negative HEK293T cells. This approach aids the development of next-generation anticancer glycovaccines by incorporating noncanonical amino acids, followed by conformational optimization and antibody-antigen complex analysis. In another study, the authors reported engineered glycopeptide nanoparticles as a diagnostic tool to detect circulating tumor-associated autoantibodies against MUC1, generally found in the serum of pancreatic cancer patients [254]. Among glycopeptides displaying GST*A epitope, nanoparticle probe bearing an N-propionylated Tn glycopeptide emerged as the most effective probe for screening pancreatic cancer patients’ serum with 85% sensitivity and 90% specificity, outperforming current clinical biomarkers CA19-9 and carcinoembryonic antigen (CEA).

Liao and coworkers reported the synthesis of a three-component glycovaccine composed of Thomsen- Friedenreich (TF) antigen, an iNKT cell agonist and Mincle ligand, and investigated its immunomodulatory response in Balb/c mice model [255]. The study describes the synthesis of TF glycopeptide fragment, derivative of iNKT cell agonist, KRN7000 and derivative of a Mincle agonist, vizantin. These fragments were later assembled utilizing amide linkage and click chemistry to construct tricomponent glycoconjugate, for example KRN7000-TF-vizantin, two-component glycoconjugate such as TF-KRN7000 and TF-vizantin. Among these vaccine candidates, KRN7000-TF-vizantin elicited a robust production of TF-specific IgG and IgM antibodies compared to TF-KLH/alum and the corresponding two-component conjugate vaccines. Further, analysis of antibody isotypes and secreted cytokines revealed that KRN7000-TF-vizantin induced both Th1 and Th2 immune responses, wherein Th1 was prominent. Additionally, KRN7000-TF-vizantin exhibited strong binding affinity for both CD1d and Mincle receptors, implying activation of iNKT and Mincle signaling pathways. The antisera from vaccinated mice displayed specific affinity along with complement-dependent cytotoxicity (CDC) towards TF-positive cancer cells. Tumor challenge studies using murine mammary carcinoma cells (TA3Ha) demonstrated that mice immunized with KRN7000-TF-vizantin exhibited improved endurance and protection against tumor rechallenge without the need for further treatment. To further explore the structure−immunogenicity relationships, the authors constructed Tn-functionalized glycoconjugates employing Mincle agonists both as a carrier and self-adjuvant [256]. The study comprised extensive chemical manipulations using chemoselective and orthogonal reactions to vary the position and number of Tn antigens and lipids on trehalose-based framework of the Mincle agonists. Immunological studies revealed that while all the conjugates prompted immune responses, the conjugate containing two Tn antigens conjugated to trehalose via an aryl linker with a lipid chain, showed the highest immunogenic response. It showed robust IgG antibody production, promoted Th1/Th2 cytokine release, and exhibited specific affinity to human Mincle-Fc protein. The extracted sera from immunized mice showed specific binding to T-positive cancer cell lines along with enhanced CDC activity. Co-administration of the conjugate with EcMPLA (a TLR4 agonist) and aluminum hydroxide (Al) further enhanced its immunogenic potency and prolonged the survival duration of mice by inhibiting the tumor growth. Additionally, upon rechallenging the surviving mice with tumor cells, no tumor growth was observed even without additional treatment.

In a rather recent report, Zong and coworkers reported glycopeptide-protein conjugate vaccines with varying glycoforms and investigated their antitumor immunity in Balb/c mice model [257]. The study described the synthesis of four variants of 21-mer MUC1 glycopeptides having different glycoforms including Tn, T, STn, and α2,3-ST antigens using chemoenzymatic liquid-phase synthetic strategy. Glycopeptides were subsequently conjugated to CRM197 carrier protein to produce glycopeptide-protein conjugate vaccines. After vaccinating the mice with MUC1-glycopeptide-CRM197 conjugates along with various combinations of adjuvants, specific antibody production was observed. Interestingly, the combination of QS21 + MPL was identified as an effective adjuvant. Further experimentation showed that, all glycosylated MUC1 vaccines formulations exhibited strong affinity for MUC1-expressing MCF-7 cancer cells and induced complement-dependent cytotoxicity (CDC) and antibody-dependent macrophage phagocytic activity (ADCP) in vitro. Furthermore, the study demonstrated serum from vaccinated mice specifically binds to human breast cancer cells. In parallel, Pesce et al. constructed GMMA vesicles fabricated with mucin-1 Tn and sTn mimetic antigens and evaluated their immunogenic responses in triple negative breast cancer models in Balb/c mice [258]. Tn (GalNAc-α-O-Ser/Thr) and sTn (sialyl-GalNAc-α-O-Ser/Thr) mimetics were synthesized by structurally locked analogs and then conjugated to genetically modified outer membrane vesicles (GMMA) purified from Gram-negative bacteria to produce GMMA-Tn and GMMA-sTn glycoconjugates. When GMMA-Tn and GMMA-sTn formulations were exposed to CD-1 and Balb/c mice, significant production of antigen-specific antibodies such as IgG1, IgG2a, and IgG2b subclasses were detected. Pifferi et al. have further advanced their strategy and prepared clustered tetravalent conjugates using cyclopeptide scaffolds grafted on to the CRM197 carrier protein [259]. This study shed light on the importance of scaffold-mediated antigen display for improvement in epitope recognition and overall vaccine potency.

Another receptor of interest in the glycoconjugate field is Cluster of differentiation 44 (CD44) that is aberrantly glycosylated as well as dysregulated on the surface of cancer cells. CD44 is a transmembrane glycoprotein that exhibits highly variable molecular weight (~ 80 to 200 kDa) due to significant structural heterogeneity [260]. Structural heterogeneity mainly arises due to alternative splicing of its gene [261, 262] and post-translational modifications (PTMs) [263]. Glycosylation, being the most prominent PTM, further amplified the structural variability wherein highly glycosylated CD44 could reach as high as 200 kDa in molecular weight [264]. CD44 is expressed on a spectrum of normal cells, and is involved in cell-cell interactions, adhesion, and migration via its interactions with the extracellular matrix [260]. CD44 is expression is dysregulated in many types of cancer, including breast, colorectal, pancreatic, and lung [265–269]. Dysregulation of CD44 in cancers predominantly varies from its overexpression, altered isoform expression and aberrant glycosylation [270, 271]. Additionally, CD44 possesses cancer stem cell traits and it is also referred to as a “stemness” marker, being associated with tumor initiation, progression, metastasis, and therapy resistance [272–274]. These unique features make CD44 an attractive target for cancer immunotherapy [275, 276].

We have delineated the two different approaches of glycoconjugate based targeting of CD44. On the one hand, its presence as a major scaffold for several TACAs such as Tn, sTn, sLe^x^ and sLe^a^ [277, 278] is used for designing TACA mimic based vaccines. On the other hand, its overexpression on the surface of tumor cells [270] has been used for designing direct-binding-mediated immunotherapeutics. The glycoconjugates developed using the former approach are summarized in this section whereas the ones utilizing the latter approach receive their due discussion in the next section of its direct binder, namely hyaluronic acid. With the parallel advancement in antibody generation techniques via the use of synthetic glycopeptides, the development of CD44 targeted antibodies has risen again. Aasted et al. devised a monoclonal antibody, 4C8, that was selective to Tn-glycosylated epitope of the CD44v6 isoform [278]. The authors used glycosylated amino acids in a Fmoc-based solid-phase peptide synthesis strategy to synthesize the glycopeptide [GYRQTPKEDSHS(Tn)TTGTAAA]. Immunization of Balb/c mice with the KLH-conjugate of this glycopeptide, followed by hybridoma based screening resulted in the isolation of 4C8, the antibody that was proposed as a glycovaccine candidate. The authors went on to engineer this antibody to construct unique CAR-T cells that showed a broad recognition towards Tn+ tumor cells, and not only to CD44v6 expressing tumors. This study highlighted the far-reaching potential of glycopeptides in the field of cancer immunotherapy.

In a very novel approach reported by Freitas et al., the formulation of glycovaccines targeting CD44s-Tn antigen positive cancer was reported [279]. First, the authors developed a glycopeptide using 30 mer fragments of CD44s by glycosylating with Tn sugar using GalNAc-transferases. Afterwards, it was sialylated by ST6GalNAc-I enzyme for achieving the complete antigen structure. Structural analysis revealed that the glycopeptides contain mixtures of Tn and sTn, which mimics the heterogeneity on specific types of cancer cells. Following the preparation of CD44-sTn glycopeptide, it was mixed with monocyte-derived dendritic cells to evaluate immune response in C57BL/6 mice. Surprisingly, CD44-sTn glycopeptides induced significant toxicity to the dendritic cells, impeding the direct use of these glycopeptides as vaccines. To mitigate this issue, the glycopeptides were conjugated with immunostimulatory carrier proteins such as CRM197 and KLH. The CD44-sTn KLH conjugate displayed enhanced immune response only when stimulated with MPLA (adjuvant). Mice immunized with KLH-CD44-sTn, and MPLA elicited antigen-specific IgG and IgM antibodies that exhibited specific affinity towards CD44-sTn glycopeptides and CD44-sTn cancer cells. Furthermore, extracted antibodies displayed complement-dependent cytotoxicity (CDC) against cancer cells expressing CD44-sTn antigens. This study showcases a promising prototype of a cancer glycovaccine thereby opening the avenues for futuristic precision medicine in cancer immunotherapy.

A close observation of the immune responses among the two classes, glycolipids and glycoproteins, reveals noteworthy trends. While mimics of glycolipids like gangliosides stimulate NK cells and innate pathways, conjugates based on glycoproteins like Mucin-type-O-glycans work by invoking the dendritic cell mediated immune response. In case of Globo-H, literature reflects a shift from weak “self-tolerant” response in case of the natural hexasaccharide to a strong T-cell dependent response in case of a fully synthetic polysaccharide conjugated to a carrier like KLH and coupled with a strong adjuvant like QS-21. The differences in nature of immune responses by different TACA-based glycoconjugates result in different kinds of benefit in their anti-tumor benefits. Specifically, MUC1 based conjugates lead to lysis of solid tumors, Globo-H conjugates induce complement-mediated killing of tumor cells and GM2 (ganglioside) conjugates lead to prevention of micrometastasis. The most significant trend identified in the last 20 years of glycobiology is the requirement of “Signal 2”. In most cases, a TACA alone adds only Signal 1 and leads to B-cell exhaustion or transient IgM. A TACA + Carrier + Adjuvant adds a Signal 2 and creates a germinal center response, which has been harnessed to achieve anti-tumor “memory” and hence a “vaccine-like” effect.

## Non-TACA based glycoconjugates

### Monosacccharides as a targeting tool

#### Glucose

Glucose is an essential component for metabolic energy and for the biosynthesis of macromolecules in nearly all mammalian cells [280, 281]. Glucose, being hydrophilic in nature, relies on transport proteins such as sodium-glucose cotransporters (SGLTs) and glucose transporters (GLUTs) for its uptake through the hydrophobic plasma membrane [280–283]. GLUT family of proteins, encoded by the *SLC2A* gene, are expressed in various tissues, and play a significant role in transporting monosaccharides across cell membranes of eukaryotic cells [283–285]. Glucose binds to the GLUT transporters, especially GLUT1 and GLUT3 via a selective and reversible mechanism. Specific amino acid residues in its transmembrane domains allow GLUT1 and 3 to alternate between outward-facing and inward-facing conformations, thereby regulating the transport of glucose [281]. The considerably high binding affinity of GLUT1 for glucose, represented by a significantly small binding constant i.e. Km ~ 1–3 mM, ensures efficient glucose uptake even under low-glucose conditions [286, 287]. The expression of GLUT1 and GLUT3 receptors are significantly upregulated in many tumors, like lung, breast, colorectal, and glioblastoma [288–292]. Tumor cells use these receptors to meet their high metabolic demands, that originate from rapidly proliferating cells. GLUT1 acts as the key mediator of the Warburg effect, i.e. the ability of tumor cells on savage energy from glycolysis [293, 294]. Transport through GLUT1 ensures a continuous glucose influx to fuel glycolysis, nucleotide biosynthesis, and lipid metabolism [295]. This unique feature of glucose dependence led to the development of a powerful tumor diagnostics platform, [18] F-fluorodeoxyglucose positron emission tomography (FDG-PET) imaging [296–298]. Overexpression of GLUT family receptors, especially GLUT1 and GLUT3 are linked with aggressiveness, metastasis and overall poorer survival rate [299].

Many oncogenic pathways such as HIF-1α, c-Myc, and PI3K/Akt signaling are known to promote overexpression of GLUT1 [300]. Due to its cancer-specific overexpression, GLUT1 is considered as a therapeutic target in anti-cancer therapies [299, 301, 302]. There has been growing interest in developing glycoconjugates and glucose-functionalized prodrugs that target GLUT transporters for cancer therapies [32, 303, 304]. Glucose functionalized nanoparticles have also been investigated as a means of enhanced presentation of glucose for efficient interaction with the overexpressed GLUT1 transporters on cancer cells [305, 306]. In a recent review, Seeberger, Yin and coworkers have nicely summarized the development of glycoconjugates that exploit the overexpression of glucose transporters like GLUT1 in cancer cells for targeted drug delivery [307]. While many antineoplastic drugs like 5FU, oxaliplatin, paclitaxel and Adriamycin have been reported to be delivered by the GLUT1 targeting, there isn’t a report of a GLUT1 targeted glycoconjugate that exerts anti-tumor effect exclusively by eliciting the immune system. Moreover, T cells also rely on GLUT receptors, especially GLUT1, to drive clonal expansion and effector function [308–310]. This shared glucose dependence within TME fuels hostile metabolic competition, which results in glucose deprivation for tumor-infiltrating lymphocytes (TILs) eventually leading to T-cell dysfunction and immune evasion [311, 312]. GLUT1 expression has been recently reported to influence immune cell infiltration in the TME [313, 314]. Reports of immunosensitization via GLUT1 inhibition and CAR T cell activation have started surfacing [315, 316]. However, these approaches are mostly involving genetic or pharmacological modulation of GLUT1 expression or function, while not using sugar conjugates. Further research that explores the potential immunomodulatory effects of such GLUT1-targeted conjugates needs to be performed.

One fascinating application of glucose-based glycoconjugates is in the field of photodynamic therapy (PDT). On the one hand, sugar derivatization helps target the PDT agents to the cancer cells via the overexpressed GLUT receptors. On the other hand, some unique PDT agents also induce an immunogenic cell death (ICD), a form of programmed cell death that alerts the immune system to the presence of the tumor cells. ICD involves the translocation of calreticulin to the cell surface and the release of high-mobility group box 1 (HMGB1) and ATP into the extracellular matrix. Both these act as damage-associated molecular patterns (DAMPs) i.e. potent recruitment and activation signals for dendritic cells (DCs). This results in an effective “priming” of the DC’s for a strong adaptive immune response. Some of these unique conjugates were developed in the past as “third-generation PDT agents”. For example, the glucose-conjugated (tetrafluorophenyl) chlorin, or G-chlorin, exhibited a 20–50 fold increase in cytotoxicity compared to the second-generation PDT agent talaporfin sodium, while inducing immunogenic cell death (ICD) and also targeting of tumor-associated macrophages (TAM’s) [317]. Another class of glucose-directed immunomodulators is “glucosylated antibodies for checkpoint blockade”. These were developed particularly in the context of glioblastoma where expression of GLUT1 on the blood-brain barrier was used to facilitate transcytosis of anti-PD1 antibody to the tumor [318]. A strategy of “glucosylated multi-PEGylation” was used to functionalize the checkpoint blockade antibodies. Reduction-sensitive linkers were used for attaching the PEG chains so that the free functional antibody could be released in presence of the high glutathione levels found in the GBM microenvironment. Glucose conjugation has also been used for precise spatial delivery of a TLR7 agonist, imiquimod (IMQ) [319]. A combinatorial nanotherapy was also developed with doxorubicin and IMQ entrapped in micellar systems that were decorated with glucose ligands on the surface of the micelle. In the acidic TME, these micelles were shown to expose the glucose ligands and cause increased accumulation of DOX and IMQ in the cancer cells and in the TAMs (both of which overexpress GLUT1 receptor). The result was apoptosis and ICD of cancer cells and M2 to M1 polarization of TAMs i.e. a sustained anti-tumor immune response in the form of DC’s and cytotoxic T-cells.

In a recent intriguing report, Pu et al. formulated a 2-deoxyglucose (2-DG) and bismuth ferrite co-loaded gel (DBG) and demonstrated cancer piezocatalytic immunotherapy [320]. The study described the synthesis of 2D BiFeO_3_ nanosheet which was then encapsulated with 2-deoxyglucose in ROS-sensitive hydrogel that was developed by combining poly(vinyl alcohol) (PVA) with ROS-labile *N*1-(4- boronobenzyl)-*N*3-(4-boronophenyl)-*N*1, *N*1, *N*3, *N*3-tetramethylpropane-1,3-diaminium (TSPBA) linkers. Following the implantation of DBG gel in tumors, ultrasound waves induced localized generation of ROS and produced piezoelectric signals that promoted the stimulation of anticancer immunity. High level of *N*-glycans is known to impede the immune response in TME, however in this case the ultrasound-mediated released 2-deoxyglucose inhibit *N*-glycosylation, synergistically overcome the immunosuppressive microenvironment of tumors. The interesting feature of this study was that nanocatalytic immunotherapy technology of DBG and ultrasound not only inhibited tumor growth but also generated a strong immunologic memory. Table 1 includes this study as a notable candidate for GLUT1-targeted cancer immunotherapy.

**Fig. 7:**
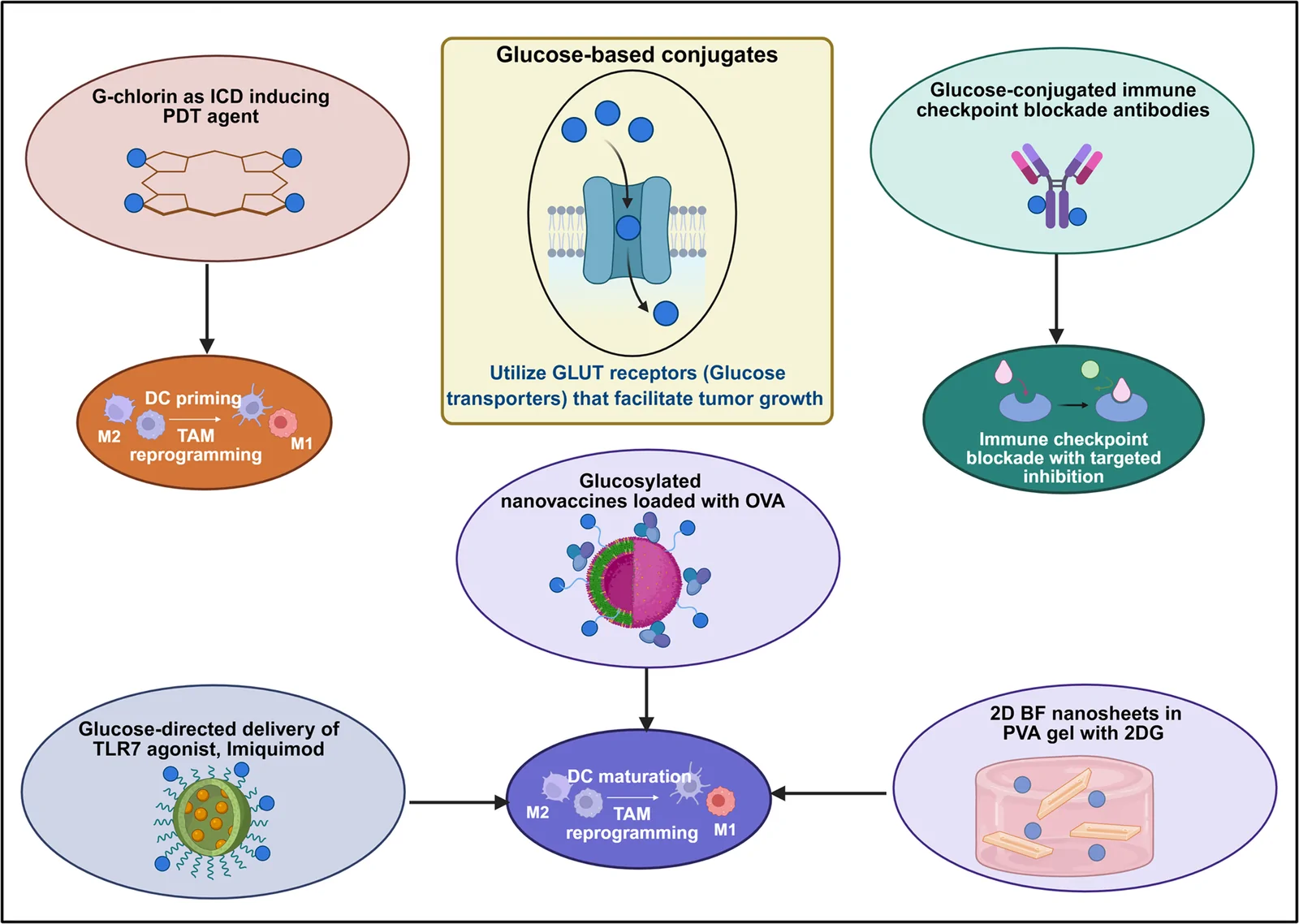
Glucose-directed conjugates for cancer immunotherapy. Targeting of GLUT receptors on tumor cells and tumor-associated DCs/TAMS has been achieved by conjugating glucose to PDT agents, TLR7 agonists, nanovaccines, nanosheets and checkpoint blockade antibodies

In line with improving anti-cancer immune memory, glucose functionalized nanovaccines were developed to reprogram DCs that are the prime orchestrators of adaptive immune response [321]. The authors confirmed that GLUT1 is a reliable target for antigen delivery to DC’s since GLUT1 is overexpressed on different kinds of DC’s and glucose consumption is critical for DC survival and activation. Glucosylated nanovaccines loaded with ovalbumin (OVA) demonstrated the ability to trigger stronger immune responses and display higher antitumor efficacy as compared to conventional non-glucosylated nanovaccines. This was attributed to the specific binding of glucose ligand to GLUT1 on the surface of DCs feeding the high glucose demands of DC activation while simultaneously promoting the presentation of the tumor associated antigen (ovalbumin) on their MHC’s. The promising candidates of immunotherapy using glucose-based conjugates are summarized in Fig. 7. Although promising, the transition of GLUT-targeted glycoconjugates from preclinical success to clinical applicability is a complex journey. The presence of GLUT1 in essential tissues like the brain and RBC’s necessitates a high degree of precision [322]. The most promising direction for these set of immunomodulators is integration with other targeting modalities to enhance specificity. Dual-targeting systems that leverage the presence of GLUT (a metabolic marker) and CD44 (a surface antigen) are being developed [323]. Moreover, the use of Class III transporters like GLUT8 that have more limited expression patterns would offer a systemically safer route for the transport of glycoconjugates.

#### Galactose

The potential of galectin-targeted strategies in cancer immunotherapy stems from the critical role these lectins play within the tumor microenvironment. Galectins are a family of soluble, β-galactoside-binding proteins that typically interact with cell-surface glycoconjugates [324]. Members of the galectin family exhibit preferential distribution across different types of tissue. Members like Gal-2, Gal-4, and Gal-7 are exclusively expressed on the body’s protective barriers i.e. the gut lining and the skin, where they manage epithelial cell turn-over and defend against pathogens [325]. Members like Gal-1 and Gal-3 are expressed across a relatively broader range of tissue types, but they preferentially ramp up inside the vasculature (blood vessels) and immune cells to control cell migration, angiogenesis (blood vessel growth), and immune tolerance [326]. While galectin expression shows a specific pattern across normal tissues, their aberrant upregulation and extracellular secretion are consistently linked to tumor immune escape and poor clinical outcomes [327–329]. In malignancies, they act as extracellular immune checkpoints, facilitating tumor progression, cell-cell interactions, and immune evasion by suppressing infiltrating T cells [330–332].

**Fig. 8:**
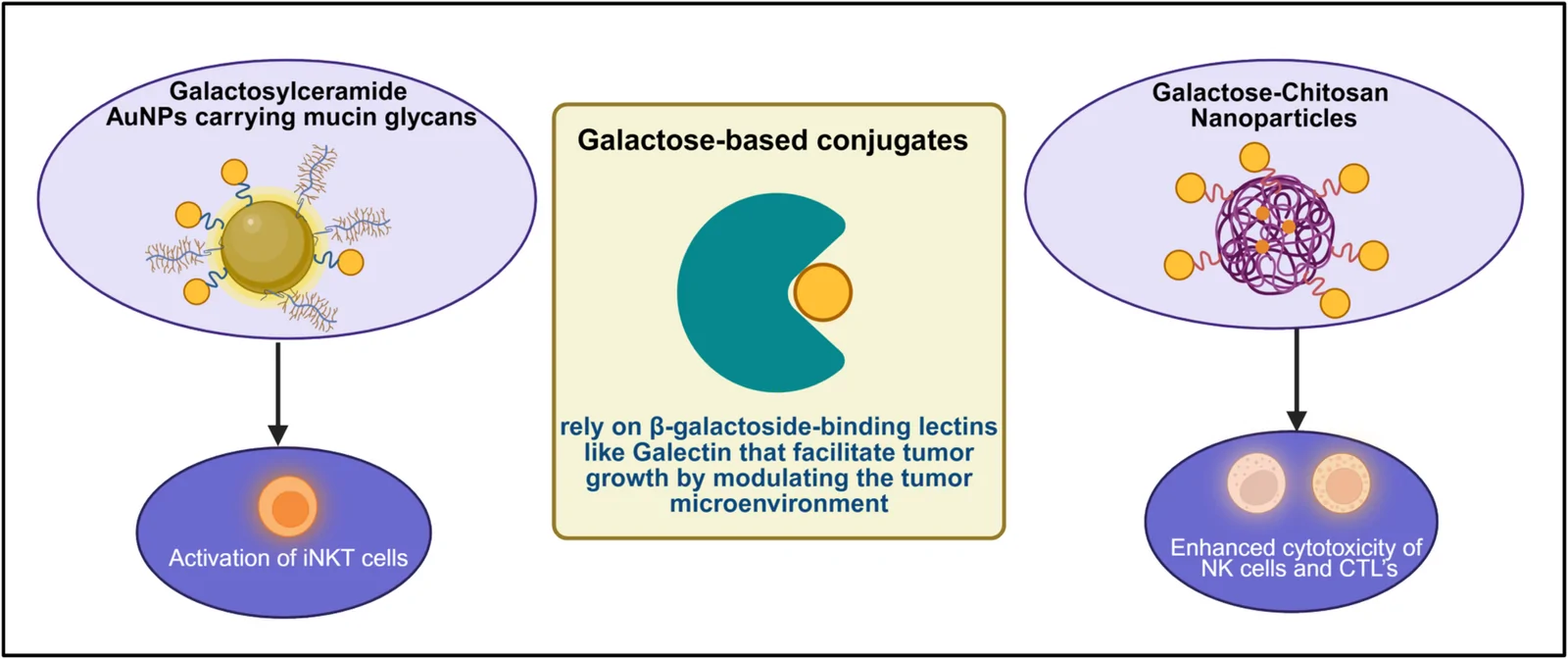
Galactose-directed conjugates for cancer immunotherapy. Galectin receptors on tumors are shown to get primed for immunogenic cell death via the use of galactose as targeting moiety. Polymeric nanoparticles as well as gold nanoparticles have been subjected to galactose functionalization for achievement of enhanced cytotoxic immune responses

Among the 15 known mammalian galectins, Galectin-1 (Gal-1) and Galectin-3 (Gal-3) are the most extensively studied [333, 334]. Gal-1 is involved in immune suppression, promoting tumor immune evasion by inducing apoptosis in activated T cells [335, 336]. Gal-3, known for its role in cancer progression, enhances tumor cell survival, angiogenesis, and metastasis by interacting with integrins and extracellular matrix (ECM) components [337]. Thus, galectins facilitate tumor growth by modulating the tumor microenvironment [338]. While they promote immune evasion, they also enhance cancer cell migration by interacting with glycoproteins such as MUC1 [339]. Additionally, galectins mediate resistance to apoptosis and contribute to chemotherapy resistance in several malignancies [340]. Due to their multifaceted roles in oncogenic advancement, numerous efforts have been made to target galectins by developing small molecules, conjugates, and macromolecular assemblies to elicit immunomodulatory response in cancers [341, 342] and the most notable ones using galactose as targeting sugar are summarized in Fig. 8.

Owing to the immunosuppressive functions of Gal-3, interest in its inhibitors as potential anticancer therapies grew with time. These inhibitors were shown to circumvent the immunosuppressive effects by disruption of the “galectin lattice” and by blocking the TREM2 (Triggering receptor expressed on myeloid cells 2) signaling on TAMs, thereby promoting M1 polarization [343]. In a very recent comprehensive review, the authors summarized a variety of galectin inhibition strategies that have shown promise at the preclinical and clinical front [344]. A significant achievement was the development of thiodigalactose (TDG), a sugar derivative that is enzymatically more resistant compared to natural disaccharides [145]. TDG is reported to decrease metastasis in breast and colon cancer models via promoting immune cells infiltrations along with reducing tumor-associated angiogenesis [346]. To enhance specificity and potency, researchers synthesized several glycomimetics of TDG, among that TD139 showed enhanced binding affinity towards Gal-3. Another excellent thio analogue of galactose monosaccharide is GB1107, which exhibits high affinity and selectivity towards Gal-3 [347]. In preclinical studies, GB1107 is shown to reduce tumor growth and limit metastases by modulating the immune response in the TME, including M2 to M1 phenotype and enhancing infiltration of CD8^+^ lymphocytes [348]. Although great advances have been made in the field of small molecule glycomimetics as galectin inhibitors, there are still important limitations regarding their specificity and pharmacokinetic profiles that have hindered their approval for clinical treatments [344, 349].

Galactose-chitosan nanoparticles (GC-NPs) have also shown promise in enhancing the specificity and effectiveness of cancer immunotherapies, particularly for liver cancers. In a notable study, researchers showed that GC-NPs delivering the drug 5-fluorouracil or 5-FU, were able to enhance the cytotoxicity of NK cells and CTL’s, contributing to a more robust anti-tumor immune response [350]. Hsu et al. demonstrated the development of galactose derived nanoparticles which elicited enhanced cancer immunogenicity in glioblastoma and pancreatic cancer [351]. Condensed glycopolymer-like nanoparticles (CG NPs) were prepared by polymerizing ortho-nitrophenyl-β-galactoside monomer through one-pot hydrothermal carbonization reaction. The CG NPs exposed galactose moieties on the surface, capable of binding to galectins on the surface of cancer and immune cells. Using orthotopic tumor models in C57BL/6 mice, the authors investigated the fate of the NP’s after internalization in cancer cells (GL261 and Panc02). CG NPs triggered ROS generation and reduced lactate efflux through blocking monocarboxylate transporter 4 (MCT-4), subsequently induced cell apoptosis. In immune cells, especially in macrophages, after internalizing via galactose receptor-mediated endocytosis, CG NPs induces M2 to M1 polarization along with upregulation of inflammatory cytokines and immune cell activation. A combination therapy of CG NPs and anti PD1/PDL1 exhibited enhanced activation of cytotoxic T lymphocytes, maturation of dendritic cells and significant tumor reduction in a mouse model. The unique photothermal properties of AuNP’s allow their use to employ NIR irradiation to induce localized hyperthermia, effectively destroying cancer cells.

These nanoparticles can be engineered to deliver immunostimulatory molecules or antigens, promoting dendritic cell maturation and subsequent activation of cytotoxic T lymphocytes. A notable example of galactose-functionalized gold nanoparticles (AuNPs) delivering an immunogenic antigen is the development of a multivalent cancer vaccine comprising the MUC1 glycopeptide antigen and the immune adjuvant α-galactosylceramide (α-GalCer) conjugated to AuNPs [352]. In this study, Zhao and coworkers synthesized gold nanoparticles functionalized with (a) the MUC1 glycopeptide i.e. a tumor-associated antigen, and (b) α-GalCer i.e. a potent activator of invariant natural killer T (iNKT) cells. The multivalent presentation of these molecules on the AuNP surface proved to enhance antigen uptake by dendritic cells and promoted a robust immune response in vitro and in vivo. Studies like this also open the avenues for more advanced combinatorial therapies like dual photothermal and immunotherapy. An overview of the various galactose derivatives that have shown promise in the field of cancer immunotherapy is presented in Table 1.

#### Mannose

As can be seen in the previously published reviews, the development of sugar-derived immunomodulators owes a lot to the growing knowledge of C-type lectin receptors or CLRs, a diverse family of carbohydrate-binding proteins that recognize fucose- and mannose-containing glycans [353, 354]. The growth in this area has led to the identification of monosaccharides as direct anti-cancer agents [355] along with the development of Lysosome-targeting chimeras (LYTACs) that utilize glycopeptide ligands containing mannose-6-phosphonate (M6Pn) for targeted pathogenic protein degradation [356, 357].

An crucial use of mannose in cancer immunotherapy is in the form of CLR targeted immunomodulators like mannose functionalized nanoparticles [358]. Mannose-functionalized nanoparticles target the CLR ]family member referred to as the mannose receptor or CD206, which is present on tumor-associated macrophages (TAMs) and dendritic cells (DCs) [359]. The targeted nanoparticles invoke a directed anti-tumor immune response in the tumor microenvironment via both mechanisms i.e. enhancing phagocytosis as well as activating antigen presentation mechanisms. The conjugated sugar moieties on the surface of nanoparticles facilitate receptor-mediated endocytosis in addition to modulating the anti-tumor immune response. When mannose was decorated on the surface of chitosan-based nanoparticles, a potential vehicle for cancer vaccine delivery was obtained [360]. These nanoparticles were able to efficiently elicit both cellular and humoral immune responses to boost the anticancer cascade. Scientists have also explored mannose functionalized PLGA nanoparticles to deliver antigens and siRNA to DC’s, which enhances antigen presentation and reprogramming of TAMS to M1 phenotype. An interesting formulation of immune adjuvant targeting micelles (M-COSA) has been used to codeliver antigen ovalbumin (OVA) and plasmid DNA encoding CCR7 (CCR7 pDNA) to the cytosol of DCs, via mannose directed targeting [361]. These micelles were found to promote DC migration to lymph nodes and boost MHC I-restricted antigen presentation.

Another study by Pei et al. showed nanoparticles composed of mannose and ovalbumin elicited MHC-I antigen presentation for cancer immunotherapy [362]. These nanoparticles were assembled via electrostatic complexing of polyethylenimine (PEI) with a glycoprotein conjugate wherein mannose functioned as DC targeting ligands and ovalbumin as the antigen for cross presentation. Upon exposure of nanoparticles to dendritic cells, a specific mannose receptor-mediated uptake which was confirmed by endosomal escape that was enabled by the proton sponge effect of PEI. Further upon cytosolic antigen delivery, dendritic cell maturation was evidenced by increasing the expression of co-stimulatory markers CD40, CD80, and CD86, along with enhanced production of inflammatory cytokines. The cross-presentation efficiency was finally demonstrated by co-culturing nanoparticles treated DCs with B3Z CD8^+^ T cell hybridomas by showing enhanced β-galactosidase activity in stimulated B3Z T cell hybridoma. Mannose-decorated liposomes (M-YL/LA-Lipo) were developed and found to effectively deliver dominant Cytotoxic T-lymphocyte (CTL) epitope peptides to DCs, thereby enhancing activation of CTLs aiding antitumor immunity [363]. The mannose modification of PEG (polyethylene glycol) on the surface of the liposome enhanced the targeted recruitment of the liposomal drug delivery system to TIDCs, proving to be a classic example of precision targeted therapy for tumors. Recently, mannose-decorated Poly(lactic-co-glycolic acid) i.e. PLGA nanoparticles were loaded with R848 (Man-pD-PLGA-NP@R848) were prepared and tested in vivo [364]. R848 is a TLR7 and 8 agonist that showed great potential but had limitations in its delivery and release into the bloodstream. The PLGA based nanoparticle system curbed the release problem of R848 by providing a targeted mechanism to enter and perform controlled release of cargo in TAMS and TIDCs for efficient tumor immunotherapy.

A recent intriguing example of a multivalent system is the mannosylated fluoropolypeptide nanovaccine (MFPCOFG) that is especially designed to work in cold tumors [365]. Being composed of mannosylated fluoropoly(D, L-cysteine) ovalbumin-loaded Fe^2+^-gallic acid, it leverages the synergistic effect of a triple antigen-metal-thermoimmunity strategy. In a murine breast cancer model, NIR irradiation causes the nanovaccine to generate strong and long-lasting immune responses. Their main mechanism of action has been attributed to promotion in the tumor infiltration of CD4^+^, CD8^+^ T-cells as well as NK cells, along with a parallel reduction in the reduction in Tregs and M2’s. As another interesting multi-component system, Song et al. recently reported the design and fabrication of an efficient glycopeptide-based nanovaccine [366]. They showed that the use of mannose-decorated polypeptide copolymers was able to target the glucose transporters and the mannose receptors, both of which are overexpressed on mature DCs, resulting in a synergistic delivery of the antigen.

**Fig. 9:**
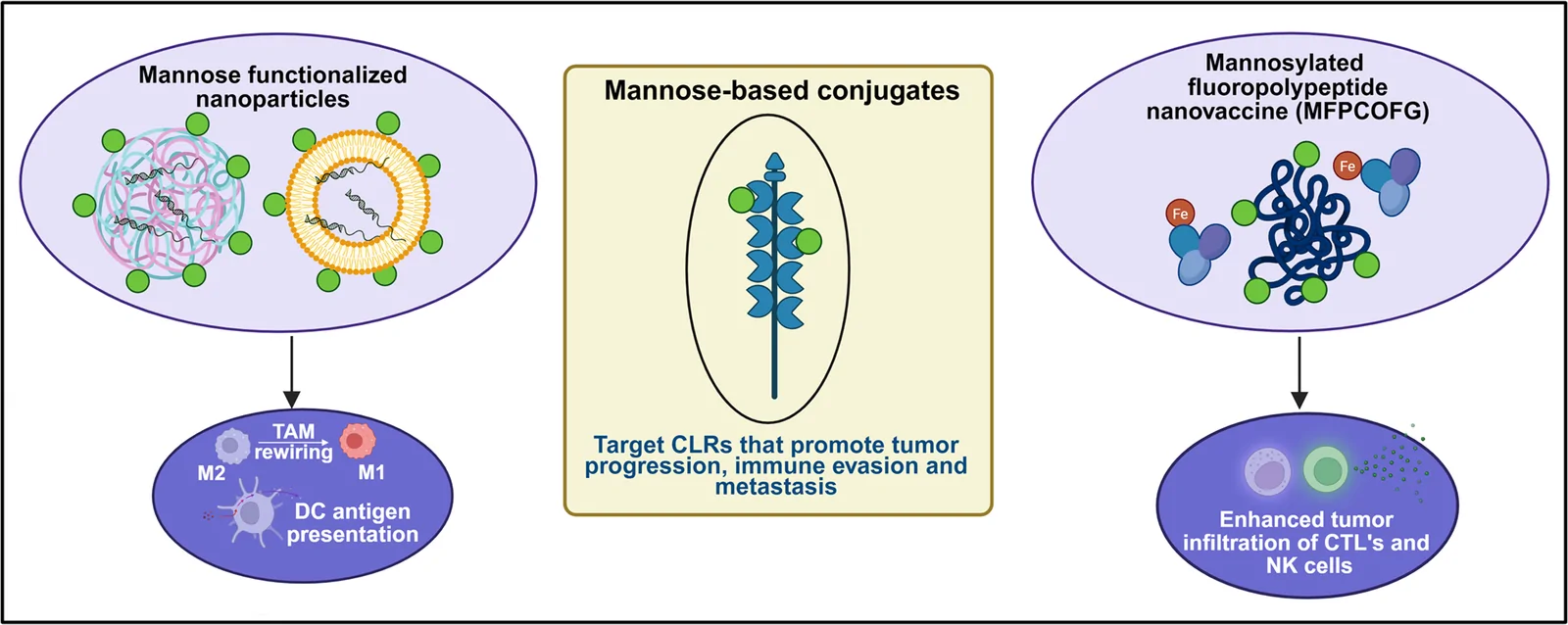
Mannose-directed conjugates for cancer immunotherapy. Mannose functionalized nanoparticles and polypeptides have been tested for CLR targeted anti-tumor immune response. Together with adjuvants like OVA, these nanovaccines have elicited increased levels of cytotoxic immune cell infiltration at the tumor sites

Very recently, Jin and colleagues demonstrated that the administration of canonical mannose sugar induces tumor suppression and regulates inflammatory microenvironment in non-small cell lung cancer (NSCLC) mice models [367]. The analysis of resected tissues, treated with mannose exhibited reduction in CD11b, Ly6G, and F4/80 expression, implying a decreased infiltration of Myeloid derived suppressor cells (MDSC’s), neutrophils, and macrophages, along with M2 to M1 macrophage polarization, while also reduced the inflammatory cytokines in mouse serum. Further analysis showed mannose treatment modulates the gut microbiota via improving probiotics and antitumor metabolites. Molecular studies confirmed that mannose treatment inhibits tumor growth in NSCLC mice models by targeting OGT/hnRNP R/JUN/IL-8 axis. A combination therapy of mannose and anti-PD-1 synergistically enhanced antitumor immunity and increased CD8^+^ T cell infiltration in TME. While Fig. 9 summarizes the existing class of mannose-based strategies proposed for CLR targeted cancer immunotherapy, the field still holds room for more extensive research and application in the multivalency format.

#### Sialic acid

Sialic acids are a family of nine-carbon sugars that are typically the terminal sugar of the glycan chains of most glycoproteins and glycolipids on mammalian cell surfaces. The aberrant upregulation of sialyltransferases results in a state of hypersialylation i.e. a dense, negatively charged layer, which acts as a physical and biochemical shield to TACAs and engaging inhibitory receptors on a wide array of immune cells [368]. Significant research has elucidated the mechanisms by which sialoglycans interact with these inhibitory receptors, called Siglecs (Sialic acid-binding Immunoglobulin-like Lectins) [77]. This family of receptors is expressed on NK cells, macrophages, neutrophils and T-cells [78–80]. The Siglec-sialic acid axis allows cancer cells to evade immune surveillance, similar to the PD-1/PD-L1 pathway [369]. As a result, sialic acid derivatives are emerging as attractive therapeutic candidates for immunotherapeutic interventions [370]. Inhibitors and antibodies designed to block Siglec-sialic acid interactions are currently in preclinical and clinical studies, aiming to restore immune cell function and enhance anti-tumor immunity [371]. Siglec-targeted antibody-drug conjugates and CAR-T cells are also being explored as a novel strategy to eradicate cancer [372–374]. The first generation of Siglec targeted glycoconjugates aimed to block the Siglec-sialic acid interactions via the use of sialic acid mimetics [375]. Researchers also modified sialoglycans conjugated to inert scaffolds for competitive inhibition against endogenous sialoglycans, with the goal of “free-ing up” the Siglecs on immune cells and restoring their anti-tumor activity [376, 377]. The focus of research then shifted towards using glycoconjugates for targeted delivery of therapeutic agents to Siglec-expressing cells [378]. This involved the use of sialylated glycans to deliver not just drugs but also immunostimulatory molecules. Taking clues from applications of inflammation [379], researchers in cancer immunotherapy advanced to the use of sialic acid decorated nanoparticles/liposomes for selective delivery of immune agonists to Siglec-expressing MSDC’s and TAMS, resulting in their reprogramming [380]. Relying on one of the most prevalent targets on the immune cells i.e. Siglec-7, -9, & -15, sialic acid functionalized nanoparticles have been used in antagonist design for immune checkpoint inhibition. In a very recent report, scientists have demonstrated that sialic acid functionalized liposomes were able to deliver arctigenin specifically to breast cancer cells leading to immunostimulation and tumor regression in a 4T1 xenograft bearing Balb/c mice model [381].

**Fig. 10:**
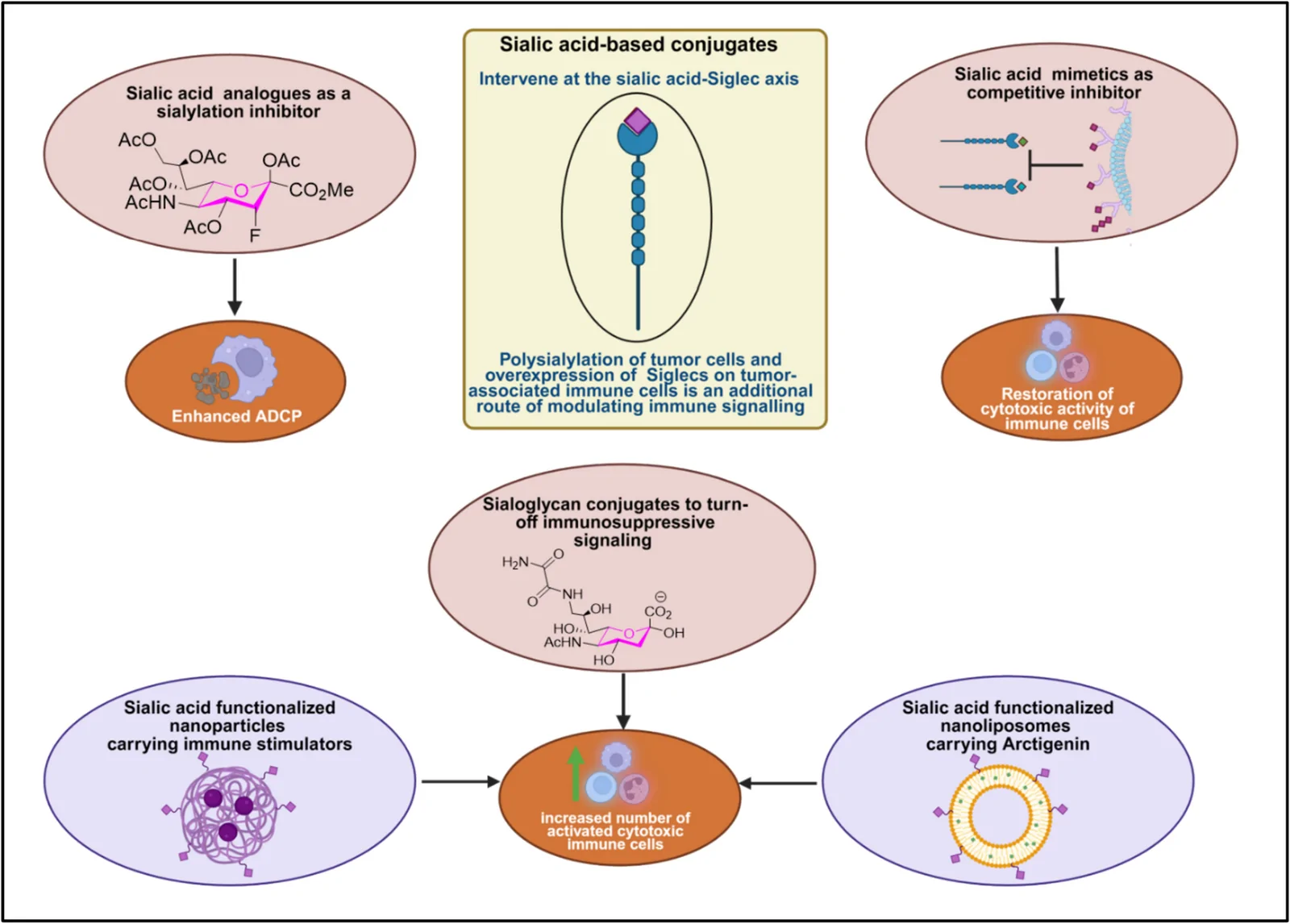
Sialic acid-directed conjugates for cancer immunotherapy. Sialic acid analogues, and mimetics have been used to intervene at the sialic-acid Siglec axis at the TME. Sialic acid functionalized nanoparticles and liposomes have demonstrated the ability to circumvent immunosuppressive signaling and promote cytotoxic immune response

In parallel, advancements in glycoengineering techniques and conjugation chemistries have enabled the generation of more sophisticated class of glycoconjugates with multivalency and controlled release properties [382]. In a very interesting report, Lustig et al. showed that a fluorinated sialic acid analogue, P-3Fax-Neu5Ac can be used as a sialyltransferase inhibitor (STi) to effect the reduction in hypersialylation on cancer cells [383]. The study demonstrated a significant reduction in sialylation on MDA-MB-468 and SK-BR-3 cells, expressing EGFR and HER2, respectively, following the treatment with P-3Fax-Neu5Ac. These STi treated cells exhibited significant Antibody Dependent Cellular Phagocytosis (ADCP) in the presence of anti-EGFR and anti-HER2 therapeutic antibodies, respectively. A moderate macrophage-dependent phagocytosis was observed when the inhibitory receptors such as Siglec-7 and Siglec-9 expressed on macrophages were blocked by their respective antibodies. Hence, the cancer cells with inhibited hypersialylation machinery exhibited enhanced macrophage-dependent phagocytosis and enhanced sensitivity to therapeutic antibodies. The key findings reported to use Sialic acid derivatives for cancer immunotherapy have been categorically summarized in Fig. 10. Although the field is still largely in preclinical stages (Table 1), the development of sialyl-glycoconjugates represents a rational and targeted approach to overcome immunosuppression and holds promise in cancer medicine.

In a recent report, Aspord and coworkers explored the influence of altered glycans present on melanoma on characteristics of human dendritic cells [384]. The study discussed exposure of neoglycoproteins (NeoGPs) consisting of BSA functionalized with glycans aberrantly found in melanoma tumors, namely Gal, Man, GalNAc, s-Tn, Fuc, and GlcNAc motifs to human PanDCs, a mixture of cDC1s, cDC2s, and pDCs. Distinctive binding profiles were observed among DC subsets based on the glycan motifs of neoglycoproteins. Interestingly, fucose-functionalized neoglycoproteins directly remodeled the expression of activation markers such as CD40, CD80.

and CD86, additionally it reshaped the immune checkpoint by upregulating expression of ICOS-L and PD-L1 in DCs. Fuc NeoGPs also altered the cytokine/chemokine secretion profile in dendritic cells. Furthermore, NeoGPs were shown to interfere with TLR signaling, priming the DCs to suppress anti-tumor cytokine responses upon subsequent TLR activation. Additionally, NeoGPs binds with C-type lectin receptors such as MGL and CLEC2, present on naïve dendritic cells. Anti-CLR blocking antibodies were shown to interfere with glycan-CLR binding, partially restoring DC functionality. This study demonstrated that a variety of monosaccharides could be exploited as potential inhibitors of the CLR/glycan checkpoint, facilitating the development of unique immunotherapy strategies

### Polysaccharides

#### Hyaluronic acid as a targeting tool

Hyaluronic acid (HA) or hyaluronan is one of the strongest binders of CD44 and is used as a targeting entity because CD44 is overexpressed in numerous malignancies [33, 385]. This affinity-guided targeting strategy aimed to improve efficacy and reduce off-target effects. Specific binding of CD44 with hyaluronic acid activates the PI3K/Akt and Ras/MAPK cascades, promoting epithelial-mesenchymal transition (EMT), self-renewal, and chemoresistance [272]. Hyaluronic acid binds to CD44 at the specialized Hyaluronan-Binding Domain (HABD) at the N-terminus of CD44 protein [386, 387]. This domain contains a “link module” that forms a shallow groove specifically shaped to cradle a 6–8-mer segment of HA. Owing to HA’s natural affinity for CD44, targeting CD44 using hyaluronic acid has been a conventional strategy in cancer therapy to enhance drug accumulation in tumors [388]. In the context of immunotherapy, the CD44 mediated tumor cell recognition of HA has been used to deliver specific immune cell agonists to aid in building up a strong anti-tumor response.

Very recently, Kuai and co-workers reported a very novel design of hyaluronic acid crosslinked lipid hybrid nanoparticles [389]. Using a single dose of these hybrid nanoparticles carrying STING (Stimulator of Interferon Genes) agonists could eliminate established colorectal tumors (MC38) in C57BL/6 mice by eliciting effective antitumor immune response. While the hyaluronic acid ensured recruitment of the multilamellar lipid hybrid NPs to the tumor site, the cGAMP released in the TME activate the STING pathway to produce increased amounts of IFNβ followed by a strong antitumor immune response. A recent study by Catania et al. led to the use of hyaluronic acid conjugates for the development of a local chemo-immunotherapy for glioblastoma [390]. They used Doxorubicin (DOX) as an immunogenic cell death inducer and CpG as a TLR-9 agonist. The combination of HA-DOX and HA-CpG lead to a cascade of immune responses in an orthotopic GL261 glioblastoma model. M2-like microglia were converted to M1 type, pro-inflammatory cytokine levels were elevated, and CD8^+^ T cell response was stimulated. As a result, nearly two-thirds of the mice treated by the combination achieved long-term survival as opposed to only one case of long-term survival out of those who received either of the monotherapies. In another study reported for melanoma, Préat and co-workers demonstrated HA-based nanovaccines delivering two different melanoma specific antigens, TRP2 and Gp100 [391]. In a preclinical setting, their composite system led to enhanced antigen presentation by DCs coupled with strong CD8^+^ T-cell responses resulting in suppression of tumor growth and prolongation of survival. A recent review has summarized many hyaluronic acid-directed lipid nanocarriers [392] and one noteworthy example relevant to the current discussion is the discovery of HABN or HA-Bilirubin Nanoparticles [393]. These NP’s were found to home to tumor cells and tumor-associated myeloid cells and deliver SC144, a small-molecule inhibitor of STAT3 pathway. This resulted in modulation of the M2-type

**Fig. 11:**
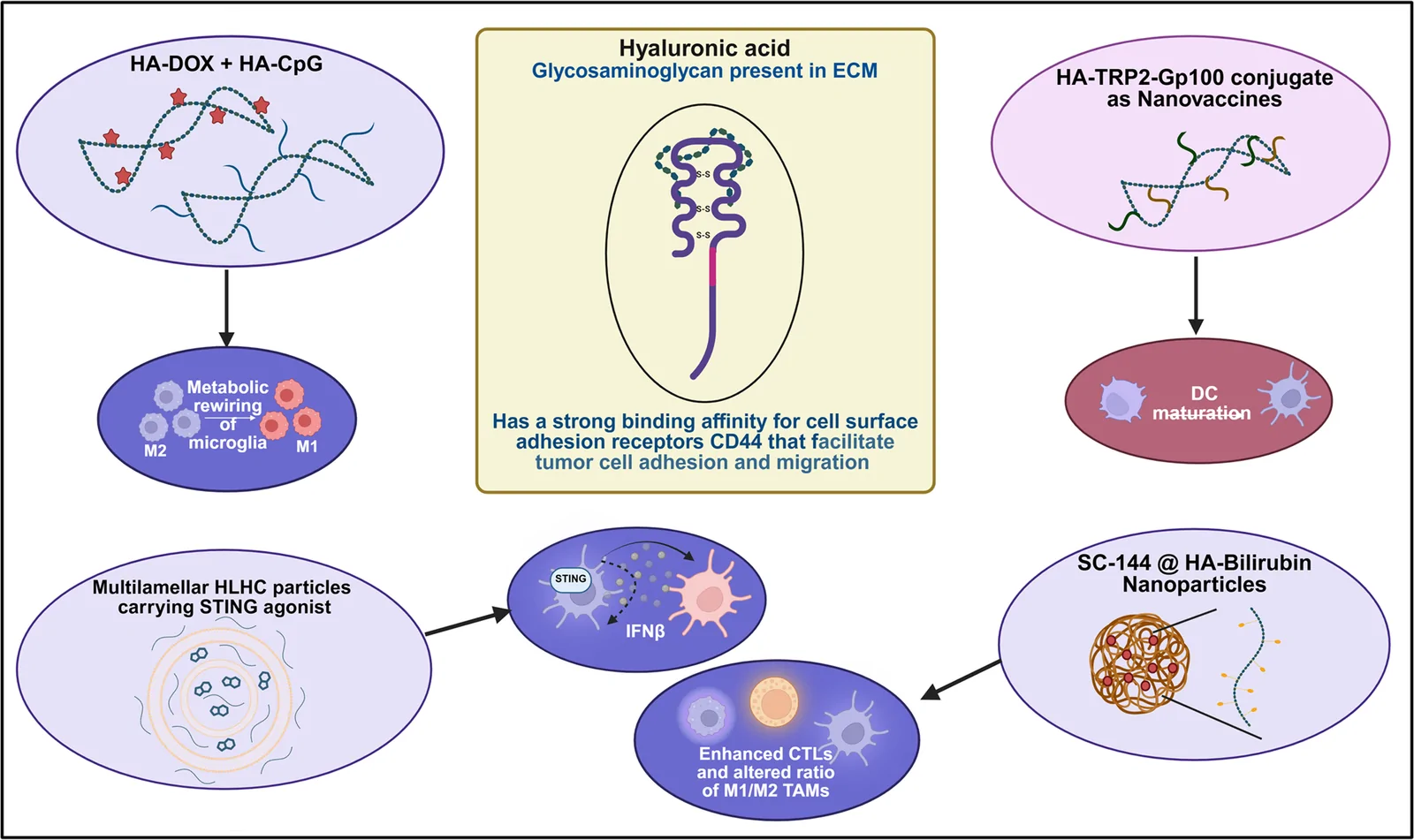
Hyaluronan-directed conjugates for cancer immunotherapy. Nanoparticles and polymeric assemblies of hyaluronan have been functionalized for delivery of immune agonists via CD44-mediated tumor targeting. Melanoma specific antigens have been effectively delivered using HA-based nanovaccines

TAMCs to a less immunosuppressive phenotype in vivo, along with triggering immunogenic cell death via enhancement of CD8^+^ T-cells. More recently, research has also expanded to the development of small molecule inhibitors targeting CD44 signaling pathways to elicit immune response [394] and CD44-targeted CAR T cells [395]. However, most of the sugar-based immunotherapy targeting CD44 remained centered around HA (Fig. 11).

Recently, Du et al. designed a cascade nanoplatform, Ce6/Pt@PDA-HA (PPCH) composed of multicomponent such as hyaluronic acid (HA), chlorin e6 (Ce6), platinum (Pt) nanozymes, and polydopamine to enhance sonodynamic therapy (SDT) for the treatment of solid tumors [396]. The study describes the preparation and characterization of spherical and stable dispersion of PPCH, furthermore establishes CD44-targeted cellular uptake and mitochondrial localization of PPCH. In vitro studies showed that PPCH induced M2 to M1 phenotype mediated by HA, this phenotypic shift prompted endogenous H₂O₂ production, which was catalytically broken down to O_2_ by platinum nanozymes, alleviating the hypoxic conditions in tumor-like environment. Subsequently, with the help of accumulated Chlorin e6 (Ce6) sonosensitizer in mitochondria, low-intensity focused ultrasound (LIFU) prompted the production of significant amount of reactive oxygen species (ROS) which ultimately triggers cancer cell killing. Additionally, PPCH was demonstrated to exhibit dual-modal imaging capacities including fluorescence and photoacoustic imaging for precise tumor diagnosis. The combination of PPCH and LIFU was shown to significantly inhibit the tumor growth in 4T1 tumor bearing Balb/c mice without any observable toxicity by modulating reversing immunosuppressive and hypoxia environment.

As another intriguing example highlighted in Table 1, Ju and coworkers developed a triply enhanced immunotherapy drug (TEID) via multicomponent integration including glycans and carbohydrate-active enzymes to enhance anticancer activity [397]. TEID was designed to achieve tumor cell membrane perforation via streptolysin O (SLO), a pore-forming toxin protein, and integration of galactose (Gal) residue and neuraminidase (NEU) to tumor cell membrane to activate NK cells and additionally to cleave sialic acids to diminish immune suppression, respectively. The study discussed the construction of SLO-Gal and SLO-NEU conjugates and demonstrated their functional activities in vitro. Further, hyaluronic acid (HA), a CD44 receptor targeting glycans, was utilized to encapsulate SLO-Gal and SLO-NEU conjugates. In vitro studies revealed the immune mediated lysis when TIED treated tumor cells were incubated with NK cells wherein SLO was validated to enhance cytokine delivery by perforating the tumor cells. In vivo studies, using 4T1 tumor bearing Balb/c mice, clearly showed that the mice treated with TEID exhibited significant tumor inhibition compared to control, highlighting the immunotherapeutic potential of perforation-assisted glycan remodeling in cancer.

#### Other immunomodulatory polysaccharides

**Fig. 12:**
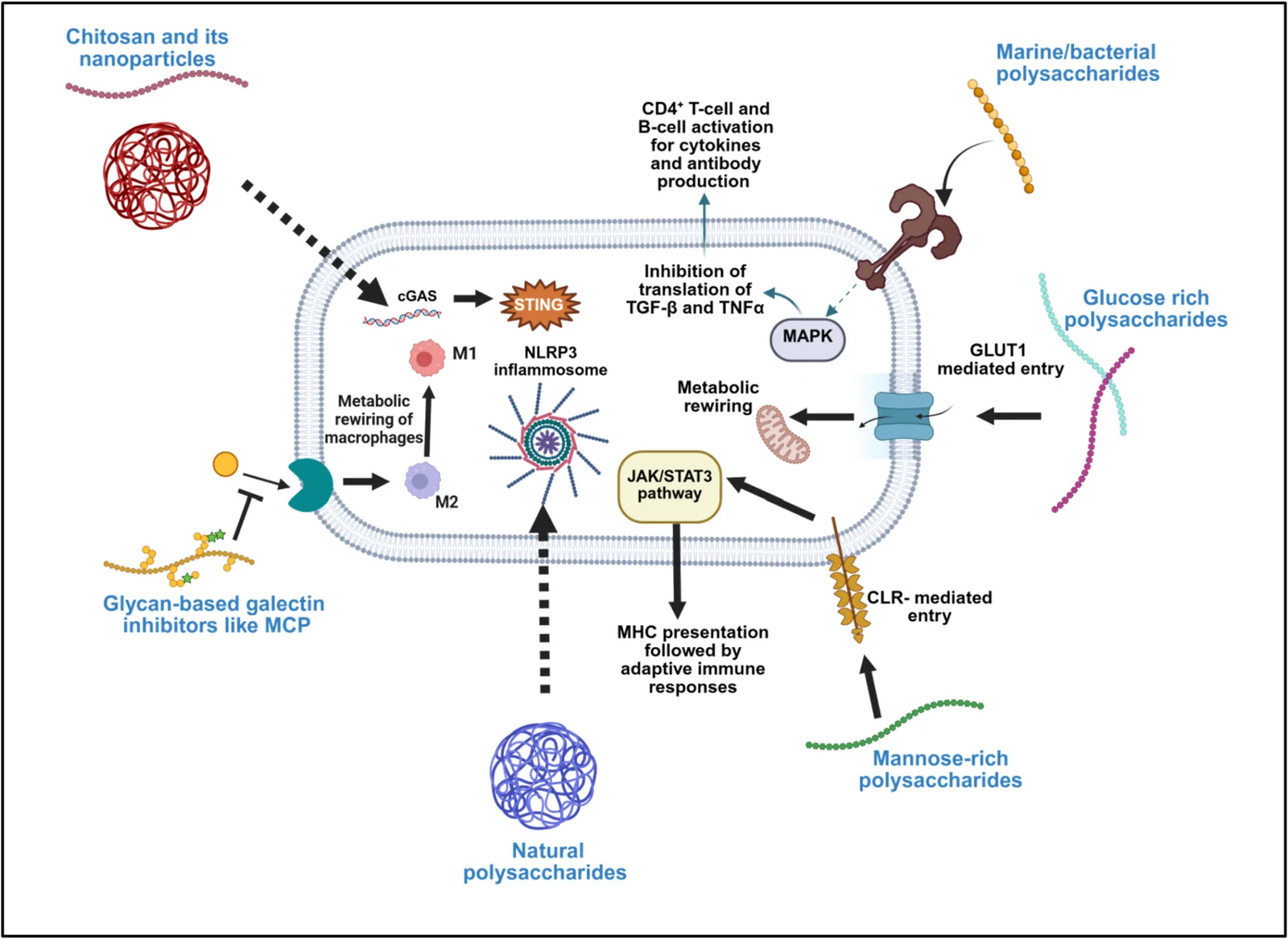
Immunomodulatory effects induced by natural polysaccharides in anticancer therapy. Different polysaccharides have been shown to modulate the STING pathway, activate the NLRP3 inflammasome, act as TLR agonists, stimulate CLR-mediated immune responses, or engage metabolic pathways to promote M1 macrophage polarization

Naturally-occurring complex carbohydrates derived from plants and microorganisms have recently garnered attention in the field of cancer immunotherapy, both as adjuvants and stand-alone agents [398]. Owing to their immunomodulatory properties coupled with their biocompatibility and low toxicity, these compounds have also shown potential to be used as nanoparticle-based approaches in immunotherapy of several types of cancers [399]. Mechanistic investigations have pointed to the ability of these polysaccharides to enhance the maturation and function of DCs, resulting in improved antigen presentation and T-cell activation [400]. Some specific polysaccharides have also shown inherent anti-cancer properties along with abilities to directly act on lymphocytes and neutrophils [401]. We describe below some of the key developments in this area and the most notable examples of how such simple systems display extremely beneficial outcomes (Fig. 12).

As the field of galectin inhibitors progressed, pectins became popular among researchers due to their biocompatibility and ease of availability from natural sources [402]. GM-CT-01 (Davanat) is one such pectin-based polysaccharide, capable of inhibiting galectins, has been also evaluated. GM-CT-01 is reported to boost the cytotoxic properties of T cells in vitro studies [403, 404]. However, Davanat and related pectin samples were refuted to bind efficiently to the canonical galectin carbohydrate-binding site and cause inhibitory effects [405]. Despite the ambiguity in the mechanism of action, the observed promising physiological effects continued to encourage scientists to study pectins for immunomodulation in cancer. Another pectin-based Gal-3 inhibitor is galactoarabinorhamnogalacturonan-rich polysaccharide called belapectin (GR-MD-02), produced via chemical processing from apple pectin [406]. GR-MD-02 exhibits synergistic effects with anti-OX40 treatment to suppress tumor growth and enhances overall survival in various syngeneic tumor challenge models in female C57BL/6 and Balb/c mice [407]. This synergistic effect is mediated through a CD8^+^ T cell-dependent mechanism and reduction in M-MDSC-meditated immune suppression. GR-MD-02 is also evaluated in combination with immune checkpoint inhibitors such as anti-PD-1 and anti-CTLA-4 antibodies in various cancer types. One of the most studied inhibitors among pectin-based polysaccharides is modified citrus pectin or MCP that has shown reduction in tumor aggression via inhibitory binding to galectin-3 through galactoside residues [408]. Interestingly, MCP treatment was shown to induce metabolic reprogramming of tumor-associated macrophages, resulting in decrease of both, tumor growth and metastasis [409, 410].

Guo and coworkers extracted polysaccharide, IJP70-1, from the flowers of a traditional medicinal plant, *Inula japonica* and observed that it could induce anticancer activity in a zebrafish model through immune modulation [411]. Homogeneous polysaccharide, IJP70-1, was mainly composed of arabinose, glucose, and galactose residues, with a molecular weight of 1.019 × 10⁵ Da, where monosaccharide units were connected via specified glycosidic linkages. Experimental data from zebrafish revealed that IJP70-1 enhances the expression of CD40, CD80, CD86, and MHC-II on RAW264.7 macrophages and DC2.4 dendritic cells. Molecular mechanism studies concluded that IJP70-1 directly binds to TLR-4 and PD-1 receptors, suggesting that it might function through TLR-4 mediated immune activation and might also function as a PD-1 inhibitor to overcome tumor immune escape, respectively. Additionally, IJP70-1 exhibited anti-angiogenesis activity by significantly inhibiting intersegmental vessel (ISV) formation through interaction with vascular endothelial growth factor (VEGF). Soon afterward, Dong et al. reported that *L. deliciosus* polysaccharide (LDP) elicits immune activation and tumor suppression through regulating intestinal flora metabolism [412]. Upon oral administration, LDP significantly suppressed solid tumor growth with an inhibitory rate of 51.61% at a high dose (50 mg/kg and 100 mg/kg) in male Balb/c mice bearing S180 tumor (sarcoma). Additionally, the study also described that LDP administration increased beneficial intestinal flora such as Lactobacillus and induced adenine-mediated zeatin biosynthesis, which competitively antagonized A2A receptor signaling and subsequently enhanced the activation of certain subsets of T cells such as CD4⁺ and CD8⁺ T cells. Finally, lymphocyte subset analysis and proteomics assays demonstrated activated T cell-mediated immunogenic cell death of tumor cells.

Another interesting example is RRTP80–1, a homogeneous polysaccharide from Rosa roxburghii [413]. Treatment of A549 tumor with RRTP80–1 in zebrafish xenograft model led to significant suppression of tumor growth and migration. Additionally, RRTP80–1 treatment upregulated the production of reactive oxygen species (ROS) and nitric oxide (NO) in a zebrafish model, these findings indicates that tumor inhibition occured primarily because of immune activation. Liu et al. reported microwave-extracted clam polysaccharide (MCP) from *Ruditapes philippinarum* exhibits antitumor activity through its dual functions as an immune-modulator and as an antioxidant [414]. Analysis revealed that the clam-derived polysaccharide was mainly composed of glucose, mannose, arabinose, rhamnose, glucuronic acid, and galacturonic acid. While MCP treatment induced downregulation of Bcl-2 and upregulation of cytochrome C and caspase-3, MCP also triggered macrophage activation by promoting M1 polarization and enhanced the macrophage-mediated cytotoxicity toward cancer cells. Additionally, MCP was also found to significantly reduce intracellular ROS levels within cancer cells and improve the anti-tumor immune response without obvious cytotoxicity towards healthy macrophages.

An investigation by Guo and coworkers revealed that a homogeneous polysaccharide, FOBP90-1 from medicinal macrofungus *Fomitopsis officinalis* could activate monocyte-derived dendritic cells and macrophages in a zebrafish model [415]. They confirmed the elevated nitric oxide production, increased phagocytic activity along with overexpression of surface maturation markers such as CD40, MHC-II, CD80, and CD86. Additionally, FOBP90-1 showed interactions with multiple key receptors including TLR-2, TLR-4, PD-L1, and VEGFR-2 suggesting its multi-target mechanism of action involving immune modulation and inhibition of angiogenesis. In a follow-up study, the authors also prepared pH-sensitive nanoparticles using immunomodulatory polysaccharide (PCP), the cross-linker 1,4-phenylenebisboronic acid BDBA and a small-molecule chemotherapeutic drug, esculetin to study antitumor and immunostimulatory effects [416]. Upon treatment of A549 cancer cells with EL@PCP-BDBA NPs, EL@PCP-BDBA displayed enhanced cytotoxicity compared with esculetin alone. Interestingly, EL@PCP-BDBA NPs exhibited a pH-responsive release behavior mimicking the tumor microenvironment. After administering EL@PCP-BDBA NPs to zebrafish models bearing tumors, an enhancement in the generation of NO and ROS was observed demonstrating stimulation of the innate immune system alongside superior anticancer activity. The study also showed that the PCP component within the nanoparticle boosted the maturation of dendritic cells as confirmed by elevated expression of maturation markers.

In another study, Guo and colleagues extracted a polysaccharide PGP40-2B from an edible and medicinal plant, *Platycodon grandiflorum* and demonstrated that PGP40-2B possesses anti-cancer immunity through immune modulation and anti-angiogenesis properties [417]. PGP40-2B isolated from *P. grandiflorum* roots is composed of rhamnose, galacturonic acid, glucose, galactose, and arabinose with a mass ratio of 1.00: 9.15: 0.82: 0.86:2.86. PGP40-2B treatment in zebrafish models bearing A549 tumor significantly reduced tumor proliferation and metastasis. PGP40-2B exhibits affinity towards vascular endothelial growth factor (VEGF). To evaluate its immunoregulatory capability, PGP40-2B was tested in immune-cell assays. An increase in production of NO and enhanced expression levels of cell co-stimulatory molecules such as CD40, CD80, CD86, and MHC-II were observed when exposed to DC2.4 cells and RAW264.7 macrophages. Additionally, PGP40-2B was also found to interact with PD-1, implying that it might also serve as an immune checkpoint inhibitor to revive T cell-orchestrated anti-tumor activity. The authors also isolated and purified a polysaccharide, JYP70-1 from the fruits of *Rosa laevigata* and investigated its antitumor activity [418]. Analytic data revealed JYP70-1 is composed of arabinose, galactose, glucose and mannose, in a molar ratio of 4.11:3.42:1.24:1.00 and it exhibits a compact coil structure in NaCl solution. Like the previous study, JYP70-1 was also found to activate DC2.4 dendritic cells. In zebrafish xenograft models, JYP70–1 significantly suppressed tumor growth and metastasis.

Luo et al. demonstrated that a natural polysaccharide from areca nut led to increased secretion of inflammatory cytokines such as TNF-α and IL-6 and enhanced production of NO, implying a pro-inflammatory M1 macrophage phenotype [419]. Additionally, the authors also observed that ANP-treated macrophages activated NF-κB and MAPK pathways, as evidenced by enhanced phosphorylation levels of p65, IκBα, p38, JNK, and ERK. Further, the study also confirmed that ANP does not bind to TLR2 and TLR4 receptors as ANP-induced M1 polarization remains unaffected by TLR2- and TLR4- specific antibodies. On the other hand, endocytosis inhibitors significantly reduced cytokine secretion by inhibiting ANP internalization.

Very recently, Li et al. investigated the potential influence of polysaccharides, extracted from *Stichopus japonicus*, on immunomodulatory functions when combined with anti-PD1 antibody in a tumor bearing mouse model [420]. Oral administration of these Sea Cucumber Polysaccharides or SCP’s in C57BL/6 mice bearing MC38 tumors with or without anti-PD1 antibody, led to an amplified anti-tumor activity. Verification by flow cytometry and immunohistological staining, showing significant enhancement in cytotoxic CD8^+^ T cells population along with reduction in regulatory Foxp3^+^ CD4^+^ T cells (Tregs). Further studies revealed that SCPs alone augmented beneficial gut microbial species and enhanced the levels of certain metabolites like indole-3-carboxylic acid which correlated with improved anticancer activity. The study also discussed that the mice receiving microbiota from SCP-treated donors showed inhibited tumor growth and improved immune profiles, supporting the conclusion that SCPs exert their effect in a microbiota-dependent manner. In conclusion, natural polysaccharides offer a unique and multifaceted approach to cancer immunotherapy.

CD73 is an ectoenzyme overexpressed in various tumors, including breast cancer. It contributes to immunosuppression within the tumor microenvironment by generating adenosine, which inhibits anti-tumor immune responses. Recently, CD73-specific siRNA was encapsulated within chitosan-lactate nanoparticles to facilitate targeted delivery and gene silencing in a 4T1 tumor bearing Balb/c mice model [421]. Using sugar-targeted gene delivery vehicles ensures tumor-localized delivery of the cargo, thereby bolstering the efficacy of the immunotherapeutic strategy. Natural polysaccharides offer a major advantage in terms of biocompatibility, especially in the context of biocompatibility and bioavailability. Different classes of derivatives have shown different mechanism if eliciting antitumor immunity, as summarized in Fig. 12. The diversity in their mechanisms of action and a highly favorable safety profile make them promising candidates for integration into current cancer treatment regimens.

## Conclusions

The altered glycosylation patterns on tumor cells confer them the ability to be distinguished from healthy cells. The unique glycosylation feature aids the tumor cells during immune surveillance and contributes to immune escape and altered oncogenic signaling pathways that lead to cancer development, progression and metastasis. Due to low immunogenicity, glycans like TACAs in their natural state are generally considered as T cell independent antigens and can only induce noncytotoxic IgM production through B cell activation. Recent technological developments such as advances in synthetic glycobiology and glycoengineering have allowed the production of glycoconjugates with carrier proteins. These conjugates can induce isotype switching to IgG as well as T-cell engagement. Due to their ability to prime both the innate and adaptive immunity, invoking immunological memory, these glycoconjugates are referred to as glycovaccines. The most successful glycovaccines have been those targeting TACAs of glycolipid origin, i.e. gangliosides and globosides (Table 1). The differential processing of glycolipid and glycoprotein-based antigens can be used to rationalize this superiority. Firstly, glycolipids bypass the MHC-based presentation and tumors frequently downregulate MHC expression. Glycolipids are expressed by CD1d molecules that are rarely mutated or downregulated by tumors. Secondly, the primary targets of CD1d-bound glycolipids are iNKT cells, which are significantly more potent than conventional CD4^+^ and CD8^+^ T cells. Despite these reasons, ganglioside-based immunotherapy has seen success only in the form of passive immunization (anti-GD2 monoclonal antibodies). Active vaccines (such as GM2-KLH) have consistently failed owing to central tolerance and high expression of gangliosides on normal neural tissues. Globo-H vaccines are the next ray of hope, especially with the GLORIA trial representing a modern dogma of patient stratification and personalized therapy. Among the glycoprotein-based vaccines, Lewis antigens are still struggling to pave their way as potential candidates. However, those using Mucin-type *O*-glycans have seen waves of successes and failures. Formulations like Theratope (sTn-KLH) emerged promising in vitro but failed at the hands of clinical microheterogeneity. Shifting from naked glycans to synthetic glycopeptides (e.g., Tn-MUC1) has afforded a structurally rigid, non-self epitope that successfully breaks central tolerance.

**Table 1:** Summary of notable examples of glycoconjugates that have shown promise in cancer immunotherapy

Glycan category	Name of the glycoconjugate	Targeting sugar moiety	Conjugating Partner	Primary effect observed	Literature/ Most recent CT ID ref.
TACA-based immunotherapeutic glycoconjugates					
Gangliosides	GD2 + GM2-KLH bivalent vaccine GM2-, GD3L-, and GD2L-KLH trivalent vaccine	GD2, GM2 and GD3 glycans	KLH, OPT-821 as adjuvant	Induction of antibodies against G(M2) and G(D2)	Chapman et al. [147] Rosenbaum et al. [154] NCT01141491
	GD2- and GD3-KLH	GD2 and GD3 glycans	KLH, β-glucan as adjuvant	Strong and robust antibody production in patients	Kushner et al. [148]/NCT00911560
	PAMAM-GD2 and PAMAM-GD3	GD2 and GD3 glycans	PAMAM dendrimers	Rapid expansion of CD8^+^ and δγ T cells	Tong et al. [151]
	GD2 and GD3 conjugates with MX1 VLPs	GD2 and GD3 glycans	bacteriophage MX1 virus-like particles (VLPs)	Induction of a high IgG titer, CDC-active sera from mice	Zhao et al. [159]
	Ganglioside-functionalized nanoliposomes	GM3 and (Neu_5_Gc)GM3	αGalCer, doped in DSPC and cholesterol liposomes	Improved CD1d presentation on DC’s, proliferation and activation of iNKT cells	Romanò et al. [160]
Globosides	GH-KLH vaccines	Synthetic Globo-H	KLH, QS-21 as adjuvant	Production of specific antibodies in vitro and in human patients	Ragupathi et al. [171] Slovin et al. [172], Wang et al. [173], Gilewski et al. [174]
	GH-DT/C34 vaccine	Synthetic Globo-H	Diphtheria toxin (DT) synthetic adjuvant α-GalCer (C34)	enhanced specificity towards breast cancer cells expressing GH, SSEA3 and SSEA4	Huang et al. [175]
	Adagloxad Simolenin (OBI-822)	Globo-H hexasaccharide	KLH, Saponin as adjuvant	Enhanced antigen presentation on MHC II, recognition by B-cells to produce GH and SSEA3-specific antibodies	Wang et al. [177] NCT03562637 [180–183]
	Globo H-CRM197 conjugate	Chemoenzymatic-ally synthesized Globo H	CRM197, Combination of adjuvants: QS-21 and 3D-MP	Strong antibody response and Th1 cellular immunity in mice	Ma et al. [184] NCT06490198
Lewis Antigens	sLe^a^-KLH conjugate	sLe^a^ glycan	KLH, QS-21 as adjuvant	Strong IgG and IgM response, highly selective MVT-5873 (human derived IgG1λ Ab)	Diab et al. [201] Gupta et al. [202] NCT03801915 NCT00470574
	sLe^a^-Qβ conjugate	sLe^a^ glycan	Qβ bacteriophage capsid	high anti-sLe^a^ IgG titers	Rashidijahanabad et al. [207]
Truncated Mucin-type O-glycans	TF antigen	TF glycan		Elicitation of cell-mediatedimmune response	Springer et al. [217, 218]
	Theratope (sTn-KLH)	sTn glycan	KLH	High antibody titers specific to synthetic STn epitope and natural mucin, OSM	MacLean et al. [230], Miles et al. [231] NCT00003638 NCT00046371
	Tecemotide (L-BLP25)	MUC1 peptide (25mer)	Palmitoyl lysine, doped in liposome (Chol, DPPC, DMPG), MPLA as adjuvant	Antigen-specific T-cell proliferation, production of IFN-γ	Sangha et al., Butts et al. [235, 236] NCT00409188
	MUC1 peptide unglycosylated	100mer synthetic MUC1 peptide	Poly-ICLC (Hiltonol)	Strong IgG response	Kimura et al. [228] NCT01720836
	Synthetic tri-component vaccine	MUC1 antigen	helper T-cell peptide saponin-as adjuvant	Strong and long-lasting antibody response	Pifferi et al. [245]
	MUC1 Tn-functionalized synthetic *S*-GalNAc glycocluster	MUC1 glycopeptides ± T helper epitope P30	tetraphenylethylene (TPE) scaffold, using α-*S*-GalNAc + amino acid linkers	Enhanced DC uptake, strong and selective antibody production	Gabba et al. [246]
	glycolipid-BSA micelle	Tn antigen	PEG-linked phospholipids, DSPE	Production of specific antibodies against Tn antigen	Thekke Veettil et al. [247]
	Tn-PV-PS A1	Tn MUC1 antigen	poliovirus (PV) peptide, tethered to PS A1 zwitterionic polysaccharide	Enhanced antigen specific T-cell proliferation and activation	Nishat et al. [251]
	Noncanonical MUC1-AuNPs (e.g. glycopeptide 1-Hnv*)	MUC1 glycopeptide containing noncanonical amino acids	Gold nanoparticles	Strong anti-MUC1 antibody response	Bermejo et al. [253]
	3-component glycovaccine (TF- antigen, an iNKT cell agonist and Vizantin)	TF-MUC1 glycopeptide fragment	iNKT cell agonist, KRN7000 and Mincle agonist, Vizantin	activation of iNKT and Mincle signaling pathways	Yang et al. [255]
	Mincle-dependent self-adjuvanted anti-Tn vaccine	2 Tn antigens	Trehalose (of vizantin) via an aryl linker with a lipid (like stearylbenzene) chain, EcMPLA as adjuvant	Strong Mincle-dependent Tn specific T cell immune responses	Li et al. [256]
	Synthetic glycopeptide-protein conjugate vaccines	Tn, T, sTn, and α2,3-ST antigens (21-mer MUC1 glycopeptide)	CRM197, QS21 + MPL as adjuvants	MUC1-specific strong CDC and ADCP effects	Teng et al. [257]
	Tn and sTn functionalized GMMA vesicles	MUC1 Tn and STn mimetic antigens	genetically modified outer membrane vesicles (GMMA) purified from Gram-negative bacteria	High titer of antigen-specific antibodies, enhanced immune cell infiltration into the TME	Pesce et al. [258]
	Sugar conjugated TLR7 agonists	MUC1 antigen	T helper epitope and CpG ODN (TLR9 agonist)	Targeting DC’s and macrophages to enhance antigen presentation and T-cell activation	Li et al. [429]
	sTn conjugated to Pam3CSK4	sTn antigen	Pam3CSK4 lipopeptide (TLR4 agonist)	High titers of IgG2a antibodies, enhanced CDC	Cai et al. [430]
	CD44-Tn glycopeptide	Tn glycan mimic	19mer CD44 peptide, KLH	Production of highly selective monoclonal antibody 4C8	Aasted et al. [278]
	C44-sTn KLH conjugate (MPLA as adjuvant)	sTn glycan mimic	30mer CD44 fragment, Conjugated to KLH	Generation of high titers of functionally active antigen-specific IgG and IgM antibodies	Freitas et al. [279]
Non-TACA based glycoconjugates					
Monosaccharides as targeting tool	G-chlorin	Glucose	Tetrafluorophenyl chlorin	Photo-induced immunogenic cell death	Kataoka et al. [317]
	Glucosylated ICB antibodies	Glucose	PEG linked anti-PD1 antibody	Immune checkpoint blockade in reduced environment of GBM	Yang et al. [318]
	Glucose functionalized nano-sized polymeric micelles	Glucose	MAA/PAA based copolymers bearing DOX	Spatially precise delivery of TLR7 agonist, Imiquimod	Wen et al. [319]
	2-deoxyglucose (2-DG)	Deoxyglucose		Piezoelectric ROS generation stimulates antitumor immunity and inhibits N-glycosylation	Pu et al. [320]
	Thiodigalactose (TDG)	Thiogalactose		Enhanced immune cell infiltration	Ito et al. [345]
	GB1107	Thiogalactose		M2 to M1 transition	Zhang et al. [347]
	GM-CT-01 (DAVANAT)	Galacturonic acid	Mannose-based polysaccharide backbone	Improved cytotoxixity of T-cells	Klyosov et al. [404]/ NCT01723813
Monosaccharides as targeting tool	Galactose-chitosan nanoparticles (GC-NPs)	Galactose	Chitosan	Enhanced cytotoxicity of NK cells and CTL’s	Cheng et al. [350]
	α-GalCer -functionalized gold nanoparticles (Au-NPs)	Galactose (as α-galactosylceramide)	Au-NP’s decorated with MUC1 glycopeptide antigen	Enhanced antigen uptake by DC’s and activated iNKT cells	Liu et al. [352]
	Mannose functionalized PLGA nanoparticles	Mannose	PLGA	Activation of TAMs and DCs	Suvarna et al. [359]
	M-COSA (immune adjuvant targeting micelles)	Mannose	ovalbumin (OVA) and plasmid DNA encoding CCR7 (CCR7 pDNA)	Promotion of DC migration to lymph nodes, enhanced MHC I antigen presentation	Yang et al. [361]
	Man-pD-PLGA-NP@R848	Mannose	PLGA nanoparticles were loaded with R848 (TLR7/8 agonist)	Targeted delivery of R848 to DC’s and controlled release	Dang et al. [364]
	Mannose-functionalized antigen nanoparticles	Mannose	Polyethylenimine (PEI) with a glycoprotein conjugate, OVA encapsulated in nanoparticle	CLR mediated endocytosis in DC’s, improved DC maturation and inflammatory cytokine release	Pei et al. [362]
	Neoglycoproteins (NeoGPs)	Gal, Man, GalNAc, s-Tn, Fuc, and GlcNAc motifs	BSA	Enhanced binding with CLRs MGL and CLEC2 on naïve DC’s, altered the cytokine/chemokine secretion profile	Niveau et al. [384]
	D-mannose	D-mannose		M2 to M1 polarization	Jin et al. [367]
	MFPCOFG (mannosylated fluoropolypeptide)	Mannose	fluoropoly(D, L-cysteine) ovalbumin-loaded Fe^2+^-gallic acid	NIR -mediated metal-thermoimmunity	He et al. [365]
	Sialic acid-mimetics (SAMs)	Sialic acid variants		Siglec inhibition by competitive binding	Büll et al. [375]
	BPC-Neu5Ac	Synthetic sialoglycan/sialosides	methyl α−9-N-(biphenyl-4-carbonyl)-amine	Siglec-2 inhibition by competitive binding (> 200 fold)	Kelm et al. [376]
	ATG@SA-Lip	Sialic acid	Octadecylamine doped in arctigenin- loaded liposomes	Tumor regression in mouse model, high immune infiltration	Liu et al. [381]
	P-3Fax-Neu5Ac	Fluorinated sialic acid analogue		Desialylation of breast cancer cells and enhanced ADCP	Lustig et al. [383]
Polysaccharides	HLHC	Hyaluronic acid (HA)	Lipid MLV carrying cGAMP	Activation of STING pathway and promotion of APC infiltration	Yu et al. [389]
	HA-DOX and HA-CpG	Hyaluronic acid (HA)	DOX, CpG (TLR9 agonist)	TLR9 activation, M2 to M1 transition, proinflammatory cytokines	Catania et al. [390]
	HA-TRP2-Gp100 nanovaccine	Hyaluronic acid (HA)	TRP2 and Gp100 (TLR2 and TLR4 agonists)	Enhanced antigen presentation by DC’s, strong CD8^+^ T-cell responses	Malfanti et al. [391]
	HABN	Hyaluronic acid (HA)	Bilirubin (encapsulating SC-144)	Inhibition of STAT3 pathway	Lee et al. [393]
	Ce6/Pt@PDA-HA (PPCH)	Hyaluronic acid (HA)	Chlorin e6 (Ce6), platinum (Pt) nanozymes, and polydopamine nanoplatform	M2 to M1 transition followed by H_2_O_2_ mediated SDT	Du et al. [396]
	Belapectin (GR-MD-02)	β-D-galactose	Polysaccharide with Rhanmose containing galactourinic acid backbone interspersed with Arabinose branches	Activation of CD8^+^ T cells and reduction of M-MDSC-mediated immune suppression	Sturgill et al. [407] NCT02575404
	Modified Citrus Pectin (MCP)	β-D-galactose	Polysaccharide with Rhanmose containing galactourinic acid backbone, some Arabinose branches	TAM reprogramming	Eliaz et al. [408]/ NCT01960946
	HA NP’s encapsulating SLO-Gal and SLO-NEU	Hyaluronan (HA), Galactose (Gal)	Streptolysin O (SLO)	Enhanced cytokine delivery and sialic acid cleavage to diminish immune suppression	Yang et al. [397]
	1V209 conjugated to Dextran	Dextran	1V209 (TLR7 agonist)	Strong Th1 biased immune responses, expansion of antigen specific CD8^+^ T cells	Shinchi et al. [431]
	1V209 conjugated to Cyclodextrin	Cyclodextrin	1V209 (TLR7 agonist)	High potencies of TNF-α and IL-6 production by DC’s (incorporation of sugar conjugates into cells via endocytosis)	Baba et al. [432]
	acGM-1.8 (polysaccharide based TLR2 agonist)	Acetylated glucomannan (DS = 1.8)		Activation of TLR2/6 heterodimer on macrophages causing M2 to M1 transition	Feng et al. [433]
	“Self-adjuvanting” cancer vaccine	TF antigen	Lipid A mimetic	TLR4 activation, enhanced TF-specific immune responses	Gao et al. [434]

After the achievement of effective anti-cancer activity using glycovaccines, additional efforts have been made to develop numerous delivery vehicles including liposomes, nanoparticles, nanocrystals, virus-like particles and 3D scaffolds to deliver anti-tumor immune-engaging therapeutics (like TLR/STING agonists) to targeted cells. The use of multivalency in glycoconjugates exemplifies the innovative strategies being developed to harness the immune system in the fight against cancer, offering hope for more effective and targeted therapies. While challenges associated with specificity, low immunogenicity and inability to produce sufficiently pure sugar material remain to be addressed, rapid innovations in synthetic platforms coupled with an evolving understating of glycoimmunology are driving this field to the forefront of anti-tumor immunotherapy in the broader battle against cancer.

### Outlooks and author perspectives

It has been observed that glycoconjugate-based strategies hold significant promise in cancer immunotherapy. However, there are four main challenges that need to be overcome for a successful clinical transition. The first and most important one is “immune tolerance” i.e., the immune system’s tolerance to self-antigens, including certain glycans [422]. This tolerance has been shown to limit the effectiveness of glycan-based vaccines in vivo, unlike the rare successful IgG engagement achieved by Dinutuximab, a chimeric mouse/human monoclonal antibody against highly dense GD2 clusters [423]. However, forcing the immune system to break central tolerance to generate high-affinity responses against normally expressed antigens can trigger severe autoimmune toxicities. This was starkly demonstrated by GD2 targeting therapies, successful antibody engagement causes intense, dose-limiting neuropathic pain, because GD2 is also expressed on peripheral nerve fibers [424]. The tolerance aspect has been overcome to some extent by using multifaceted carriers and adjuvants like α-GalCer and zwitterionic polysaccharides. The second major hurdle to be overcome is the “heterogeneity of glycosylation” and epitope masking. Since there is variability in glycosylation patterns among different tumor types and within a single tumor, it is significantly challenging to design a set of universally effective glycoconjugate therapies [425]. Preclinical models often utilize cell line-based models with homogeneous expression patterns. Conversely, human tumors exhibit a wide range of inter-patient and intra-tumor glycodiversity, including highly variable branching and dynamic truncation. The clinical outcome of single-glycoform therapeutics permits rapid immune escape and hence stands compromised. Multivalent systems have been proposed as the solution to this challenge due to their ability to enhance antigen density and diversity. However, the bioavailability and safe use of these synthetic multifaceted and multivalent systems is the next challenge to be met for clinical applicability. Additionally, while a synthetic glycan is fully accessible to an antibody in a culture dish, the target epitopes are often physically hidden or “masked” by dense extracellular matrix networks and hyper-sialylated glycocalyx within a 3D human tumor [426]. This masking renders a high antibody titer completely inert because the antibody is not able to dock on the tumor cells in the real patient. While traditionally the third key challenge faced by TACA vaccines was the lack of processing by the proteasome and MHC presentation leading to a failure to recruit a T-cell response, the use of carrier proteins appeared to solve the problem. That, however, added another problem called “carrier-induced suppression” which is currently the third key challenge to clinical translation. When small glycans are conjugated to bulky protein carriers (like KLH), the human immune system shifts its preference in response to the foreign protein rather than to the small glycan epitope. Researchers are trying to overcome this challenge using the “self-adjuvanting” vaccine design [427]. The fourth and final challenge that is yet to be overcome is the counteracting immunosuppressive tumor microenvironment and soluble antigen shedding. On the one hand, TAMs and MDSCs are continuously on the lookout to shut down ADCC by rendering effector cells inactive. Simultaneously, advanced epithelial tumors actively shed surface glycans and mucins (such as MUC1 or sLe^a^) into systemic circulation. These free-floating antigens act as “decoys,” binding and neutralizing vaccine-induced antibodies in vivo before they can ever reach the physical tumor site [428].

We believe that researchers in this field can significantly benefit from nextgen techniques for designing sophisticated therapeutic strategies. For example, advances in glycoproteomics and mass spectrometry are refining the identification of cancer-specific glycoconjugates. Moving forward, in vivo applications of CRISPR/Cas9-mediated glycoengineering can be explored to manipulate tumor glycosylation to enhance immune recognition. Similarly, the use of artificial intelligence (AI)-assisted glycan biomarker discovery can further accelerate the process of target identification, virtual screening and identification of novel glycan-based immune therapies, such as glyco-modified dendritic cell vaccines and glycan-targeting CAR-T cells. Thus, the focus of upcoming research should aim to combine these fast-paced techniques for developing personalized glycan-based therapies, and combination strategies that integrate glycoconjugate immunotherapies with other treatment modalities.

## Data Availability

No datasets were generated or analysed during the current study.
